# Characterization of *Staphylococcus intermedius* Group Isolates Associated with Animals from Antarctica and Emended Description of *Staphylococcus delphini*

**DOI:** 10.3390/microorganisms8020204

**Published:** 2020-02-01

**Authors:** Veronika Vrbovská, Ivo Sedláček, Michal Zeman, Pavel Švec, Vojtěch Kovařovic, Ondrej Šedo, Monika Laichmanová, Jiří Doškař, Roman Pantůček

**Affiliations:** 1 Division of Genetics and Molecular Biology, Department of Experimental Biology, Faculty of Science, Masaryk University, Kotlářská 2, 611 37 Brno, Czech Republic; veronika.vrbovska@gmail.com (V.V.); michal.zeman91@gmail.com (M.Z.); 408266@mail.muni.cz (V.K.); doskar@sci.muni.cz (J.D.); 2 Czech Collection of Microorganisms, Department of Experimental Biology, Faculty of Science, Masaryk University, Kamenice 5, 625 00 Brno, Czech Republic; ivo@sci.muni.cz (I.S.); mpavel@sci.muni.cz (P.Š.); monikadr@sci.muni.cz (M.L.); 3 Central European Institute of Technology, Masaryk University, Kamenice 5, 625 00 Brno, Czech Republic; sedo@sci.muni.cz

**Keywords:** *Staphylococcus delphini*, *Staphylococcus pseudintermedius*, Antarctica, mobile genetic elements, surface proteins, exfoliative toxin, Adélie penguin, Weddell seal

## Abstract

Members of the genus *Staphylococcus* are widespread in nature and occupy a variety of niches, however, staphylococcal colonization of animals in the Antarctic environment has not been adequately studied. Here, we describe the first isolation and characterization of two *Staphylococcus intermedius* group (SIG) members, *Staphylococcus delphini* and *Staphylococcus pseudintermedius,* in Antarctic wildlife. *Staphylococcus delphini* were found exclusively in Adélie penguins. The report of *S. pseudintermedius* from Weddell seals confirmed its occurrence in all families of the suborder Caniformia. Partial RNA polymerase beta-subunit (*rpoB)* gene sequencing, repetitive PCR fingerprinting with the (GTG)_5_ primer, and matrix-assisted laser-desorption/ionization time-of-flight mass spectrometry gave consistent identification results and proved to be suitable for identifying SIG members. Comparative genomics of *S. delphini* isolates revealed variable genomic elements, including new prophages, a novel phage-inducible chromosomal island, and numerous putative virulence factors. Surface and extracellular protein distribution were compared between genomes and showed strain-specific profiles. The pathogenic potential of *S. delphini* was enhanced by a novel type of exfoliative toxin, trypsin-like serine protease cluster, and enterotoxin C. Detailed analysis of phenotypic characteristics performed on six Antarctic isolates of *S. delphini* and eight reference strains from different animal sources enabled us to emend the species description of *S. delphini*.

## 1. Introduction

Staphylococci are a major group of bacteria inhabiting the skin, skin glands, and mucous membranes of humans, other mammals, and birds, as well as the environment due to their ubiquity and adaptability [1]. However, there is very limited information available on staphylococcal isolates from Antarctica. Researchers have occasionally isolated staphylococcal strains from Antarctic environmental samples [2,3,4,5,6] and also from several animals obtained during their health evaluation, but they were mostly only classified to the genus level, such as isolates from whale wound lesions [7], a fish stomach [8], lesions on two dead Adélie penguins [9], or skin swabs of Weddell seals [10].

To the best of our knowledge, no member of the *Staphylococcus intermedius* group (SIG), which includes important veterinary pathogens, has been reported so far from animals in Antarctica. Originally, most isolates of SIG were classified as *S. intermedius,* before their subdivision into SIG members [11,12] comprised of the following four closely related but distinct coagulase-positive species: *S. intermedius* [13], *S. pseudintermedius* [14], *S. delphini* [15], and a human-originated *S. cornubiensis* [16]. The phenotypic and biochemical differentiation between the members of this group is complex and difficult and can result in unreliable species identification [17]. These species can be interchangeably misidentified as each other. Since SIG species are very similar to *Staphylococcus aureus* based on their clinical manifestations and biochemical characteristics such as coagulase positivity, SIG human clinical isolates have probably been misidentified as *S. aureus* in the past [18]. Protein profiling through matrix-assisted laser-desorption/ionization time-of-flight mass spectrometry (MALDI-TOF MS) seems to be the most reliable method to differentiate between SIG species [19,20]. Other effective methods for SIG diagnostics are sequencing of the housekeeping genes *nuc* [21], *sodA,* and *hsp60* [22], and for the discrimination of *S. pseudintermedius* by means of the *pta* housekeeping gene [23] or multilocus sequence typing (MLST) [24], as well as whole-genome sequencing [25,26].

SIG members are common colonizers of animal mucosal surfaces and are considered to be opportunistic pathogens in many infections of different animal hosts. Staphylococcal isolates from dogs previously identified as *S. intermedius* belong almost exclusively to the species *S. pseudintermedius*, while true *S. intermedius* are found predominantly in pigeons [11,12]. *S. pseudintermedius* is recognized as an important causative agent of pyoderma, dermatitis, and otitis externa in dogs, cats, horses, and other animals [27]. However, its host range is not restricted only to non-human animals, but colonization and zoonotic infections of humans are increasingly recognized [28,29,30,31,32,33,34]. The taxonomic description of *S. delphini,* in 1988 [15], was based on two strains isolated from purulent skin lesions of two dolphins. Recently, developments in diagnostics have led to the detection of *S. delphini* in a broad range of animals [11,12,35] and one reported human case, probably of zoonotic origin [36]. According to the multilocus sequence analysis of the *sodA*, *hsp60,* and *nuc* genes, *S. delphini* is divided into two phylogenetically distinct clades; group A closely related to the *S. delphini* type strain, and group B related to the *S. pseudintermedius* type strain [12]. The large number of different host associations suggests that multiple ecovars can exist among *S. delphini* isolates.

The aim of this study was to describe the SIG isolates from mammals and birds on James Ross and Seymour Islands in Antarctica, with a focus on *Staphylococcus delphini*. The SIG strains were characterized in detail by phenotypic and genotypic techniques and based on the results an emended description of *S. delphini* was provided.

## 2. Materials and Methods

### 2.1. Bacterial Strains and Their Biochemical Characterization

**Figure 1:**
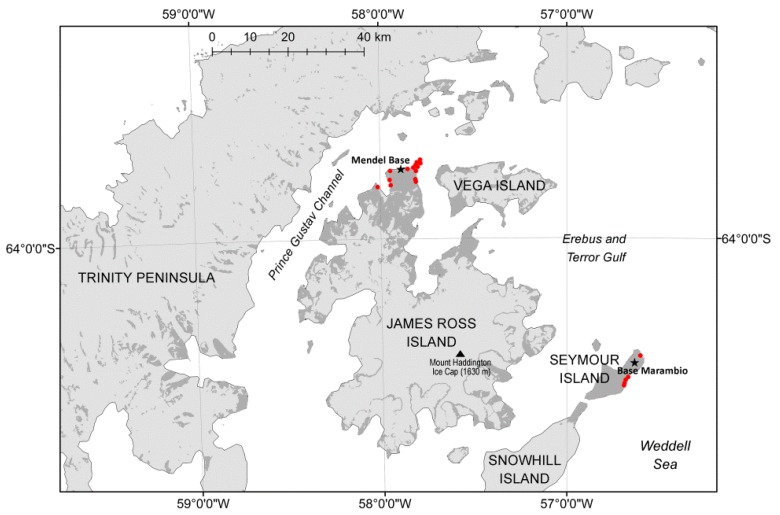
Sampling localities on James Ross Island and Seymour Island. Red mark, location of sampling site; asterisk, location of Antarctic base station; dark grey color, unglaciated area.

Bacterial strains were collected over the years from 2013 to 2019 on James Ross Island and Seymour Island, Antarctica (Figure 1). The sampling was a part of the Cultivable Fecal Bacteria Communities study, which was part of the CzechPolar project. Sampling intensity varied considerably with geographic location and weather conditions. The key element in the distribution of sampling intensity was the ice-free coast of James Ross and Seymour Islands. There are no seal colonies occupying littoral zone of both islands and samples were taken from occasionally found seals sunning on the coast. Penguin rookeries are in Seymour Island but only the droppings were collected in this territory for protection of the birds. Additional samples from penguins were taken by random sampling when penguins occurred on the coast. All samples originated from live animals only and the sampling procedure was fast to prevent any stress to the animals. The samples from the beak and cloaca of Adélie penguins (*Pygoscelis adeliae*) (82 specimens), the fresh droppings of South polar skua (*Stercorarius maccormicki*) (14 specimens), and kelp gull (*Larus dominicanus*) (11 specimens), and the anus and mouth of Weddell seals *(Leptonychotes weddellii*) (244 specimens), and Southern elephant seals (*Mirounga leonina*) (12 individuals) were collected using the swab/transport tube system E-Swab (Dispolab, Brno, Czech Republic) and cultured on Mannitol Salt Agar (HiMedia Laboratories, Mumbai, India) at 35 °C for several days in the laboratory at J.G. Mendel Base, James Ross Island. Colonies with suspected staphylococcal morphology were picked daily, kept at 4 °C and transferred to the Czech Republic for further analyses. 

Type strains of *S. delphini* CCM 4115^T^*, S. intermedius* CCM 5739^T^, *S. pseudintermedius* CCM 7315^T^*,* and *S. cornubiensis* CCM 8997^T^ were acquired from the Czech Collection of Microorganisms (Brno, Czech Republic). Reference strains of *S. delphini*: Nono (=CCM 4184) /dolphin/, CCM 2618 (=CCUG 51769) /mink/, P12548, P12549, and P12550 (=16-9169-2, 17-7762-1, and 18-3863-5, respectively) /all from minks/ [37], P12456 (=HT 2030677) /camel/, P12457 and P12458 (=8086 and 9106) /both from horses/ [11] and CCM 8998 (=MI 18-1587) /human/ [36] were described previously and kindly provided by the authors.

The phenotypic characterization of SIG strains was performed using the commercial kits API 50 CH and API ZYM (bioMérieux, Craponne, France) and by conventional physiological, biochemical, and growth tests discriminative for the genus *Staphylococcus* as described previously [2]. 

### 2.2. Partial 16S rRNA and RNA polymerase beta-subunit (rpoB) Gene Sequencing and Phylogenetic Analysis

Partial 16S rRNA gene amplification was performed as described previously [38]. PCR amplicons were sequenced with primer 553L in Eurofins Genomics sequencing facility (Ebersberg, Germany). Sequences were identified using the EzTaxon database [39].

Partial RNA polymerase β-subunit (*rpoB*) gene amplification was performed as described previously [40]. PCR amplicons obtained with the primers 1418F and 3554R were sequenced with primers 1418F and 1876R in Eurofins Genomics. Phylogenetic relationships were computed with the software MEGA version 10 [41]. Partial *rpoB* gene sequences were deposited into the GenBank/ENA/DDBJ database under accession numbers MN729216-MN729246.

### 2.3. Rep-PCR

Repetitive PCR fingerprinting with the (GTG)_5_ primer (rep-PCR) was performed as described previously [42]. Numerical analysis of the fingerprints and dendrogram construction was completed using the software BioNumerics version 7.6 (Applied Maths, Kortrijk, Belgium) and compared to the in-house Czech Collection of Microorganisms rep-PCR database of type and reference strains representing hitherto described *Staphylococcus* spp.

### 2.4. MALDI-TOF MS 

Protein fingerprints by MALDI-TOF MS were acquired with an ultrafleXtreme instrument (Bruker Daltonics, Bremen, Germany) by following the ethanol/formic acid extraction protocol [43]. As many as nine independent mass spectra were acquired for each sample, and only signals present in the minimum of seven of these mass spectra were used for the identification and cluster analysis. The mass spectral data were compared with entries in the latest version of the Biotyper database (version 9.0.0.0, 8468 references) and their mutual similarity was expressed by log(scores), where scores greater than 2.000 correlate to species identification with high confidence, log(score) values between 1.700 and 1.999 correspond to species identification with low confidence, and log(score) values lower than 1.699 result in no identification. A MALDI-TOF MS-based dendrogram was constructed with the software Biotyper (version 3.1, Bruker Daltonics) using the Pearson’s product moment similarity coefficient and the unweighted pair group method with arithmetic average (UPGMA) as a grouping method.

### 2.5. Whole Genome Sequencing and Bioinformatic Analyses

Genomic DNA was isolated using a High Pure PCR Template Preparation Kit (Roche Diagnostics, Mannheim, Germany) according to the manufacturer’s instructions with the modification of adding 20 µL of lysostaphin (0.5 mg mL^−1^) for cell lysis. An Illumina NextSeq sequencing platform was used for whole-genome shotgun sequencing of the strains P5747, P6456, and P8688. The purified genomic DNA was used for the preparation of a 500-bp sequencing library with a NEBNext® Ultra™ II DNA Library Prep Kit for Illumina (New England Biolabs, Ipswich, MA, USA). The samples were sequenced using a MID output cartridge in a 150-bp paired-end mode (Illumina, San Diego, CA, USA). The quality of sequencing reads was analyzed with FastQC version 0.11.8 [44]. Bases of lower quality and adapters were trimmed using the Spades version 3.13 implementation of BayesHammer. The de novo assembly of trimmed reads was performed with Spades version 3.13 [45], all k-mers 21 to 127 and follow-up mismatch correction. The package BBMap version 38.73 was used to analyze various statistics of genomes [46]. Contigs of described *S. delphini* genomes were reordered according to the reference genome of *S. delphini* strain NCTC 12225^T^ (GenBank accession no. LR134263, NCTC 3000 Project) by Mauve Contig Mover as a part of Mauve version 2.4 [47]. The sequences of strains P5747, P6456, and P8688 were annotated by Prokka version 1.13.7 [48]. Subcellular localization of proteins of strains was predicted using PSORTb version 3.0 [49]. Proteins assigned to extracellular or cell wall categories were classified to gene ontology by cell2go [50]. Virulence factors were predicted using the VFanalyzer tool available at the Virulence Factors Database [51]. OrthoVenn2 [52] was used to cluster predicted proteins to orthologous groups. Web-based tools PHASTER [53], CRISPR-Cas++ [54], and GView [55] were employed to find mobile genetic elements and dissimilar regions between analyzed genomes. Sequences were manipulated and examined in the cross-platform software Ugene version 1.31.1 [56]. The multiple sequence alignment was visualised using EasyFig version 2.3 [57]. 

Whole genome-based phylogenetic analysis was also carried out in order to check the species status of SIG isolates. Average nucleotide identity values (ANI) were calculated with OrthoANIu version 1.2 [58] and digital DNA–DNA hybridization (dDDH) values with the Genome-to-Genome Distance Calculator (GGDC) version 2.1 using the formula 2 recommended for draft genomes [57]. Up-to-date Bacterial Core Gene set (UBCG) software version 3.0 [59] was used to compare 92 core genes between *S. delphini* strains.

Assembly accession numbers of genomes retrieved from NCBI genome database are as follows: 8086 (GCF_000308115.1) [25], NCTC 12225^T^ (GCF_900636325.1), LMG 22219^T^ (GCF_001792775.2), NCTC 11048^T^ (GCF_900458545.1) [60], NW1^T^ (GCF_900183575.1) [16], 14S03309-1 (GCF_002374115.1), 14S03313-1 (GCF_002374125.1), 14S03318-1 (GCF_002369645.1), and 215100905101-2 (GCF_002369695.1) [26].

The Whole-Genome Shotgun projects of the *S. delphini* strains P5747 and P6456, and *S. pseudintermedius* P8688 have been deposited in DDBJ/ENA/GenBank under the accession numbers WNLD00000000, WNLE00000000, and JAACIP000000000, respectively.

## 3. Results

### 3.1. Bacterial Strain Collection and Identification of Staphylococci

During the austral summers from 2013 to 2019 on James Ross Island and Seymour Island, Antarctica, a total of 363 animal-related samples were collected in order to isolate staphylococcal species. The swabbed seals and penguins exhibited no disease symptoms at the time of sampling. Pure cultures of Gram-positive, catalase-positive cocci, able to grow in the presence of 12% NaCl, resistant to bacitracin and susceptible to furazolidone were chosen for partial 16S rRNA gene sequencing. In total, 150 *Staphylococcus* spp. strains were identified and most of them classified to the species level (Table S1). The most prevalent staphylococcal isolates were *Staphylococcus haemolyticus* (*n* = 28, predominantly in seals and droppings of skua), *Staphylococcus epidermidis* (*n* = 22, seals only), *Staphylococcus intermedius* group (*n* = 22, seals and penguins), *Staphylococcus sciuri* (*n* = 18, predominantly in penguins and droppings of skua), *Staphylococcus aureus*/*Staphylococcus argenteus*/*Staphylococcus schweitzeri* complex (*n* = 14, in all sources), *Staphylococcus capitis*/*Staphylococcus caprae* complex (*n* = 12, seals only), *Staphylococcus saprophyticus*/*Staphylococcus* *edaphicus* complex (*n* = 9, predominantly in seals), and *Staphylococcus schleiferi* subsp. *coagulans* (*n* = 6, in all sources).

Twenty-two of the 150 strains were originally identified as members of the *Staphylococcus intermedius* group based on partial 16S rRNA gene sequencing. The strains of SIG were further characterized in detail by genotypic and phenotypic methods and compared with the type strains *S. delphini* CCM 4115^T^, *S. intermedius* CCM 5739^T^, *S. pseudintermedius* CCM 7315^T^, and *S. cornubiensis* CCM 8997^T^, as well as with *S. delphini* reference strains CCM 4184, CCM 2618, P12456, P12457, P12458, P12548, P12549, P12550, and CCM 8998 isolated previously from various sources.

### 3.2. rpoB Gene Sequencing

**Figure 2:**
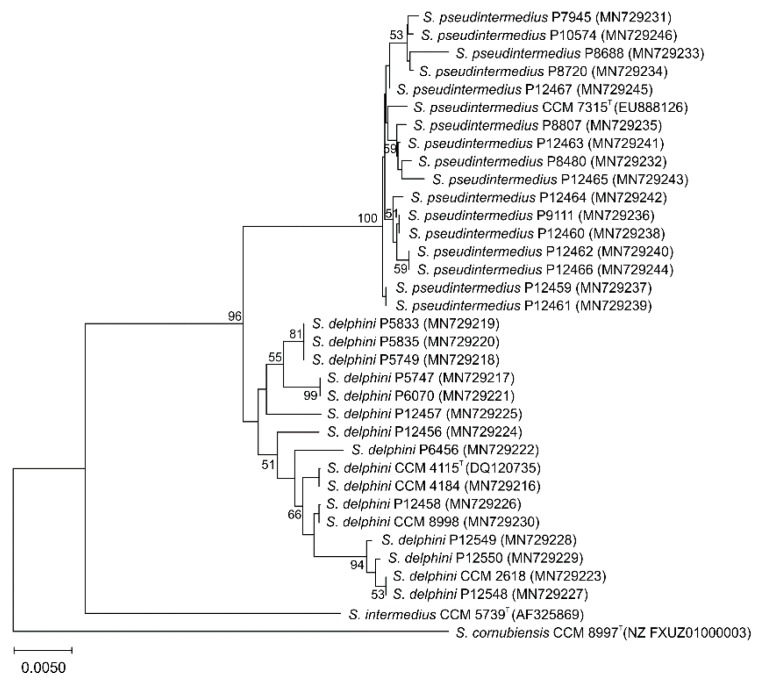
Unrooted neighbor-joining tree based on partial RNA polymerase beta-subunit (*rpoB*) gene sequence comparison, showing the clustering of Antarctic *Staphylococcus intermedius* group isolates and type and reference strains. The percentage of 500 tree replications above 50% in which the associated strains clustered together is shown next to the branches. The tree is drawn to scale, with branch lengths in the same units as those of the evolutionary distances used to infer the phylogenetic tree. The evolutionary distances were computed using the Tajima–Nei method and are in the units of the number of base substitutions per site. All ambiguous positions were removed for each sequence pair (pairwise deletion option). There was a total of 845 positions in the final dataset.

Since 16S rRNA analysis has limited discriminatory power for identifying some staphylococcal species, the phylogenetic position of SIG strains assigned by the 16S rRNA gene sequencing was assessed using the sequence data of the partial *rpoB* gene that was previously shown to be effective for the species differentiation of staphylococci [40,61]. The computed neighbor-joining phylogenetic tree based on the partial *rpoB* gene sequence showed that SIG isolates clustered into two major groups corresponding to *S. delphini* and *S. pseudintermedius* (Figure 2). Strains recovered from Adélie penguins clustered together with the *S. delphini* type strain CCM 4115^T^, whereas strains isolated from Weddell seals created a cluster with *S. pseudintermedius* type strain CCM 7315^T^.

### 3.3. Repetitive Sequence-Based PCR (Rep-PCR) Fingerprinting

**Figure 3:**
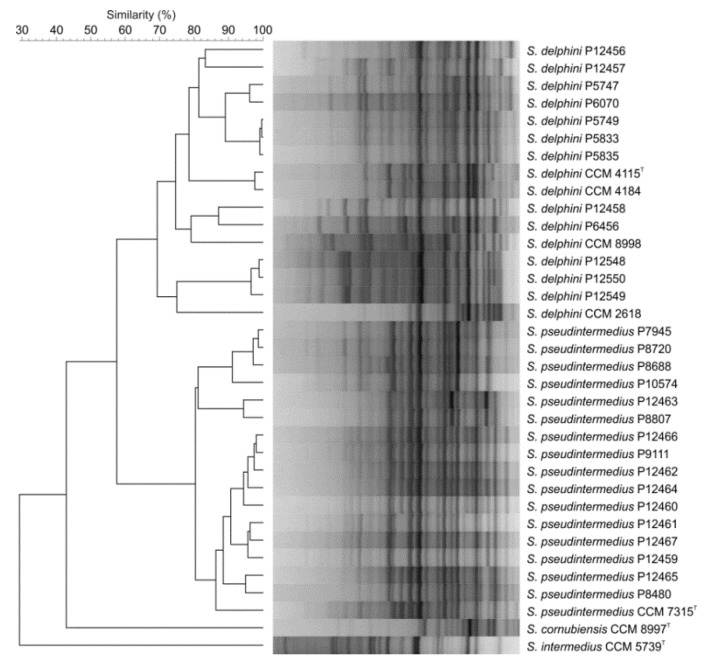
Dendrogram based on cluster analysis of repetitive sequence-based PCR (rep-PCR) fingerprints obtained with (GTG)_5_ primer from analyzed isolates and type and reference strains of *Staphylococcus intermedius* group. The dendrogram was calculated with Pearson’s correlation coefficients with unweighted pair group method with arithmetic average (UPGMA) clustering method (*r*, expressed as percentage similarity values).

The repetitive sequence-based PCR (rep-PCR) fingerprinting technique using the (GTG)_5_ primer placed the SIG isolates into two clusters corresponding to *S. delphini* and *S. pseudintermedius* at the similarity level of 54% and clearly separated them from *S. intermedius* and *S. cornubiensis* type strains (Figure 3). These results confirmed the *rpoB*-based identification of these strains. *S. delphini* strains exhibited more variable profiles (74% to 100% similarity between strains) than *S. pseudintermedius* (80% to 98%). Five *S. delphini* strains P5747, P5749, P5833, P5835, and P6070 were closely related, while strain P6456 separated from them and clustered together with the *S. delphini* P12458 strain recovered from horse nasal swab.

### 3.4. MALDI-TOF MS

All SIG isolates and reference strains were analyzed by MALDI-TOF MS fingerprinting. Out of the *S. delphini* strains involved in the study, 14 were assigned to *S. delphini* entries of the Biotyper database (six with high confidence and eight with low confidence), while two strains, P5747 and P6070, did not exhibit significant similarity to any of the database entries. All sixteen *S. delphini* strains shared several signals (3787, 4111, 4278, 5302, 6241, 6341, 7438, 8032, 8147, 9028, 9649, and 10506 Da; detected doubly protonated forms of these proteins are not listed), while no peaks characteristic of the strain origin (isolation source/region) were found.

**Figure 4:**
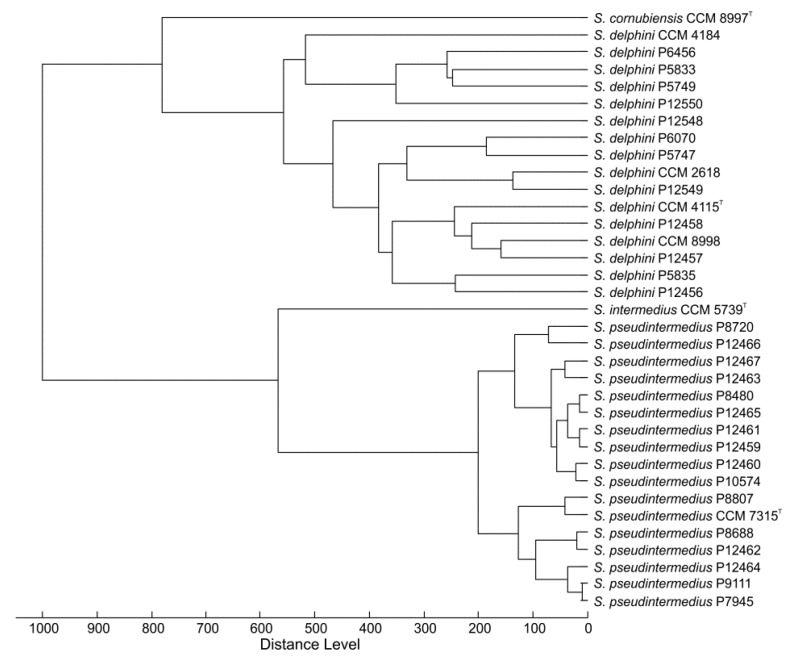
Matrix-assisted laser-desorption/ionization time-of-flight (MALDI-TOF) mass spectra-based dendrogram of analyzed isolates and type and reference strains of *Staphylococcus intermedius* group. The dendrogram was constructed using Pearson’s product moment coefficient as a measure of similarity and the unweighted pair group average linked method (UPGMA) as a grouping method.

As for *S. pseudintermedius* strains, 13 strains were identified accordingly by using the Biotyper Database (two with high confidence and 11 with low confidence), while the four remaining strains (P7945, P8807, P9111, and P12464) did not exhibit significant similarity to any of the database entries. All 17 *S. pseudintermedius* strains shared several peaks (4278, 5303, 5843, 6241, 6875, 7438, 8032, 8147, 8622, 8649, 9028, 10081, and 10506 Da; detected doubly protonated forms of these proteins are not listed). Seven signals shared by all strains of *S. delphini* and *S. pseudintermedius* (4278, 6241, 7438, 8032, 8147, 9028, and 10506 Da) indicate the close relatedness of these two species. MALDI-TOF MS-based identification outputs correlated with the dendrogram derived from the protein signals (Figure 4). In addition, two *S. delphini* strains, P5747 and P6070, and four *S. pseudintermedius* strains, P7945, P8807, P9111, and P12464 unassigned to *S. delphini* or *S. pseudintermedius* by the MALDI MS-based scoring identification workflow and grouped using cluster analysis (Figure 4) in agreement with the *rpoB*-based and rep-PCR identification (Figure 3).

### 3.5. Biochemical Identification

The physiological and biochemical properties of SIG Antarctic isolates and 13 strains isolated from other sources were examined (Table 1). Antarctic isolates of both *S. delphini* and *S. pseudintermedius* phenotypically do not correspond to previously described profiles of the species and did not allow their correct classification. Contrary to the original description, all the studied *S. delphini* strains were gelatinase positive, but lecithinase negative and produced acid from trehalose. Coagulase, arginine dihydrolase, urease, hydrolysis of Tween 80, acid production from glycerol, mannitol, β-gentiobiose, or turanose were strain-dependent for *S. delphini* (Table 1). Similarly, *S. pseudintermedius* from seals differs from the valid description by negative acetoin production and gave variable results for coagulase, arginine dihydrolase, and growth at 45 °C and acid production from mannitol, α-methyl-D-glucoside, and turanose (Table 1). Species from SIG are closely related and phenotypic classification to the species level remains insufficient due to the already reported lack of distinguishable biochemical tests for correct and reliable identification [11,12]. Phenotypic tests applicable for the presumptive species identification of SIG members based on our study, especially for *S. delphini* and *S. pseudintermedius*, are summarized in Table 2. 

All strains were positive for the following conventional tests: production of catalase, growth in 12% NaCl, bacitracin resistance, furazolidone and novobiocin susceptibility, production of DNase, pyrrolidonyl arylamidase, nitrate reduction, and growth at 37 °C. Negative for production of oxidase, starch hydrolysis, and growth at 20 °C and 48 °C, production of ornithine decarboxylases, and Voges-Proskauer (acetoin). With the API ZYM kit, positive reactions were obtained for the enzymatic activity of alkaline phosphatase, acid phosphatase, esterase (C4), esterase-lipase (C8), leucine arylamidase and β-galactosidase; and negative reactions for the enzymes lipase (C14), valine arylamidase, cystine arylamidase, trypsin, α-chymotrypsin, naphthol-AS-BI-phosphohydrolase, α-galactosidase, β-glucuronidase, α-glucosidase, β-glucosidase, N-acetyl-β-glucosaminidase, α-mannosidase, and α-fucosidase. In the API 50 CH tests, all strains gave positive reactions for acid production from glycerol, ribose, galactose, glucose, fructose, mannose, N-acetyl-glucosamine, maltose, lactose, and sucrose. Negative acid production from erythritol, D-arabinose, L-arabinose, D-xylose, L-xylose adonitol, β-methyl-D-xyloside, sorbose, rhamnose, dulcitol, inositol, sorbitol, α-methyl-D-mannoside, amygdalin, arbutin, salicin, celobiose, melibiose, inulin, melezitose, raffinose, starch, glycogen, xylitol, D-lyxose, D-tagatose, D-fucose, L-fucose, D-arabitol, L-arabitol, D-gluconate, 2-keto-gluconate, and 5-keto-gluconate; and negative esculin hydrolysis.

### 3.6. Comparative Genomic Analysis of Staphylococcus delphini Isolates

**Figure 5:**
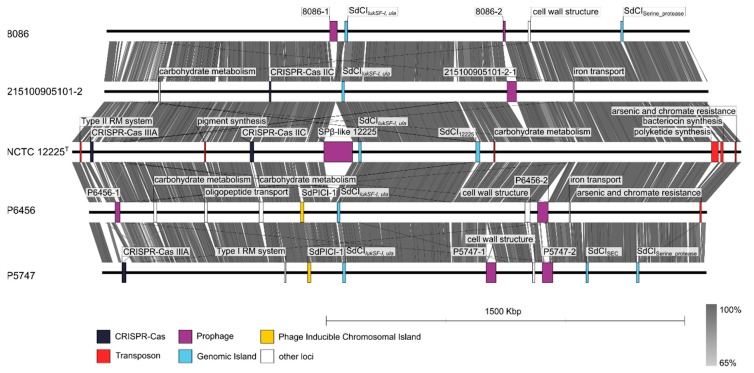
Whole-genome comparison of *Staphylococcus delphini* penguin isolates P5747 (GenBank accession no. WNLD00000000) and P6456 (WNLE00000000) with horse strains 8086 (CAIA00000000), 215100905101-2 (MWUT00000000), and type strain NCTC 12225^T^ from a dolphin (LR134263). Genomic regions with major differences between neighboring genomes and significant mobile genetic elements are highlighted in rectangles and are color-coded according to the legend. Prophage genomes 8086-1, 8086-2, and P6456-1 were located on different contigs, and therefore are not complete in the whole-genome comparison. Conserved regions with more than 65% homology are indicated with different shades of grey as determined by blastn.

Two genomes of *S. delphini* Antarctic strains that differed from each other in several characteristics were shotgun sequenced and annotated. Strain P5747 was not successfully identified by the MALDI-TOF MS scoring algorithm, was urease negative, and did not produce acid from lactose; strain P6456 formed a distant cluster in the rep-PCR based dendrogram and *rpoB* gene phylogenetic tree and did not produce acid from mannitol. The size of the draft genomes of strains P5747 and P6456 was 2.54 and 2.65 Mb, comprised of 47 and 104 contigs, with an average G+C content of 38.2 and 38.1 mol%, respectively (Table S2). The genome assemblies were compared to whole-genome sequences of *S. delphini* type strain NCTC 12225^T^ and horse isolates *S. delphini* 8086 [25] and *S. delphini* 215100905101-2 [26] (Figure 5). Comparative genomic analysis of predicted genes in the above five genomes identified 2460 gene clusters and 596 singletons. The majority of the orthologous clusters (up to 1899) were shared by all the analyzed strains. Fifty-six clusters were unique for Antarctic strains and responsible for plasmid maintenance, phage DNA replication, and a response to phosphate starvation. All non-Antarctic *S. delphini* strains have additional genes for biofilm formation, the regulation of L-carnitine utilization, and a phosphoenolpyruvate-dependent sugar phosphotransferase system. Antarctic *S. delphini* lack the presence of the genes that confer resistance to fosfomycin as compared with the strains of equine origin. 

Variability in *S. delphini* genome structure is mostly associated with mobile genetic elements and numerous genomic islets (Table S2 and Figure 5). The genomes of *S. delphini* strains P5747 and P6456 contain predicted plasmid contigs, prophages, a novel *S. delphini* phage-inducible chromosomal island (PICI) designated SdPICI-1 inserted between the chaperone GroEL and glucosamine-6-phosphate N-acetyltransferase genes, and genomic island designated SdCI_SEC_ encoding staphylococcal enterotoxin C (SEC) and putative proteins involved in its transfer. *Staphylococcus delphini* strain P5747 harbours CRISPR type IIIA, a distinctive gene cluster for purine metabolism, genes encoding sphingomyelinase C, sialidase B, putative genes for capsular polysaccharide synthesis located near the *oriC* region, and trypsin-like serine protease gene cluster. A genomic island containing bi-component leukocidin locus *lukSF-I* and the L-ascorbate transport and utilization (*ula*) operon was observed in all the analyzed genomes. The genome of *S. delphini* strain P6456 contains a gene for a novel exfoliative toxin (locus tag: FMF08_11915) in the *oriC* environ, gene clusters for the intake and metabolism of saccharides and oligopeptides, and a composite transposon for arsenic and chromate resistance.

The genes for virulence factors, surface and extracellular proteins are dispersed throughout *S. delphini* genomes. Majority of these proteins have similar distribution to other SIG members (Table S3). The comparative analysis showed three major differences in those proteins from *S. delphini* strains and representative SIG members originating from different hosts as follows: (i) variability of serine proteases including exfoliative toxin-like proteins, (ii) different polysaccharide capsule synthesis proteins, and (iii) extensive variability of putative proteins with unknown function containing a LPXTG motif anchoring to cell wall peptidoglycan (Table S3).

**Figure 6:**
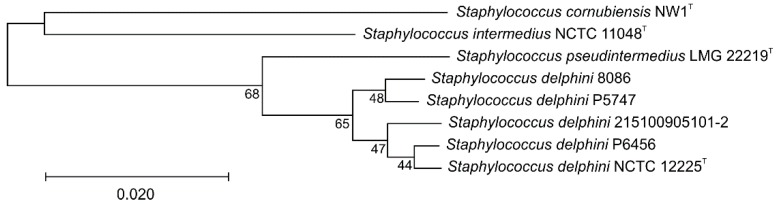
Core gene set phylogenetic tree of *Staphylococcus delphini* strains from different animals (penguin isolates P5747 and P6456, horse strains 8086 and 215100905101-2, and a dolphin strain NCTC 12225^T^). The phylogenetic tree was constructed using Up-to-date bacterial core gene set (UBCG) concatenated alignment of 92 core genes. A total of 86,031 nucleotide positions were used. Maximum likelihood phylogenetic tree was inferred using Fasttree version 2.1.10 using GTR + CAT model. Gene support indices are given at branching points (maximal possible value is 92). Bar, 0.1 substitution per position.

To evaluate the intergenomic distances between the *S. delphini* genomes, ANI and dDDH values were determined (Table 3). The calculated ANI values among analyzed *S. delphini* genomes ranged from 96.28% to 98.66%, and unambiguously confirmed the position of both strains P5747 and P6456 as *S. delphini* species and at the same time showed that *S. pseudintermedius* and *S. intermedius* type strains are well below the thresholds of 95% to 96% for species delineation [62]. The calculated dDDH values among *S. delphini* were relatively low and indicated an extensive variability that could represent specialization to specific hosts. As a further extension to genome-based phylogeny the Up-to-date Bacterial Core Gene (UBCG) tool was used to compare 92 core genes among analyzed *S. delphini* genomes (Figure 6). This analysis confirmed that the strains do not divide to distinctive lineages according to their animal host.

## 4. Discussion

The analyzed SIG strains were collected in several consecutive years at three different locations. Species identification based on previously evaluated 16S ribosomal DNA-based identification [63] showed the presence of 16 staphylococcal taxa. Some of the recently described staphylococcal species shared high similarity in their 16S rRNA sequence and were distinguished on the basis of whole-genome sequencing, therefore they are identified as species complexes. Among the most prevalent staphylococcal species isolated from Antarctic wildlife were *S. haemolyticus,* *S. epidermidis,* and *S. sciuri*, which have been previously collected in Antarctic environmental samples [4,5,6,64]. In this study, several strains of *S. aureus* were also collected, which corresponds with the findings of Van Elk et al. [65], who recovered an *S. aureus* isolate from Antarctic southern elephant seal and suggested that it could be a host species-specific and coevolved variant which is not closely related to *S. aureus* strains from terrestrial species, based on MLST analysis.

To the best of our knowledge, this is the first report of the isolation and characterization of *S. delphini* and *S. pseudintermedius* from Antarctic animals, penguins and seals. SIG species, thus, seem to be ubiquitous and the most common coagulase-positive staphylococci recovered from non-human animals, in which they can act as opportunistic pathogens and cause a variety of infections. *Staphylococcus delphini* has been previously isolated from a broad range of phylogenetically unrelated mammals (dolphins, cows, horses, camels, and mustelids). The isolation of *S. delphini* from the Antarctic Adélie penguin is the only report from birds except for rare isolates from pigeons [12]. *S. pseudintermedius* is a predominant opportunistic pathogen of canine hosts, and the clonal diversity and broad geographic distribution of *S. pseudintermedius* suggests that it has coevolved with the suborder Caniformia ("dog-like" carnivorans) for a long time in evolutionary terms [11,66,67]. Aarestrup [67] isolated SIG strains from the skin of healthy members of six phylogenetic groups within Caniformia, but not successfully from the seal family (*Phocidae*). The report of the occurrence of *S. pseudintermedius* in Weddell seals supports the theory of *S. pseudintermedius* coevolution with Caniformia, and it is evident that the bacteria can coexist with their hosts in the extreme polar environment. It is likely that SIG species shared a common ancestor and evolved with their specific host adaptation, analogously to the relationship between *S. aureus* and its sister primate-associated species *Staphylococcus simiae* [68,69] or *Staphylococcus schweitzeri* [70]. In addition, this study concluded that members of SIG are distributed worldwide and inhabit an even wider range of hosts than was previously thought.

Differentiating between species belonging to SIG is problematic. In addition, some strains previously described as *S. intermedius* have been reassigned to other species within SIG [12,71]. Identification based on conventional biochemical, physiological, and growth tests in a routine microbiology laboratory is difficult, since SIG members share many phenotypic characteristics, the expression of many biochemical properties is variable, and taxonomic descriptions were done with various identification sets utilizing different reaction substrates. We evaluated the performance of molecular-based and phenotype-based techniques for the ability to differentiate and identify members of SIG, including Antarctic isolates. The study demonstrated that *rpoB* gene sequencing and rep-PCR gave consistent results and differentiated the isolates into distinctive clusters corresponding to individual species within SIG. Cluster analysis based on MALDI-TOF MS protein data was able to separate the *S. pseudintermedius* and *S. delphini* Antarctic isolates, place them together with corresponding type strains, and create separate clusters from *S. intermedius* and *S. cornubiensis* type strains. Unlike a recent report [72], mass spectrometry was unable to identify two Antarctic isolates of *S. delphini* using a MALDI-TOF MS-based scoring identification workflow.

The Antarctic *S. delphini* and *S. pseudintermedius* isolates phenotypically differ from both the type and used reference strains. The type strain of *S. delphini* is described as negative for trehalose and thermonuclease. Because the species description of *S. delphini* was based on only two strains, in our study we tested a set of Antarctic isolates supplemented with *S. delphini* reference strains from different animals, including a human isolate. All Antarctic *S. delphini* strains were positive for acid production from trehalose, similarly to Sasaki et al. [12] who also reported some *S. delphini* strains to be positive for trehalose and thermonuclease. Moreover, in our study we found positive gelatinase activity among *S. delphini* isolates and reference strains, which contradicted their species description, as well as lecithinase production, which was positive for the type strain but negative for the tested strains. It is possible that strains from diverse animals represent distinctive ecotypes with specific ecological adaptations. Variation in phenotype, borderline dDDH values between strains (Table 3), and previously reported polymorphisms in housekeeping genes [12] suggest that more subspecies exists within *S. delphini* species.

Similar to *S. aureus* [73,74], the major differences in the *S. delphini* genomes are due to variable genetic elements. Various prophages with low similarity to known phage sequences in available databases were found in all strains. Two different types of wall teichoic acids synthesis loci were found in the analyzed genomes. Since wall teichoic acids serve as receptors for bacteriophage adsorption [74] and the phage adsorption is sufficient for efficient transduction [75] we hypothesize that differences in phage receptors can drive the diversification of *S. delphini* genomes. The staphylococcal cassette chromosome *mec,* which was described in methicillin-resistant *S. pseudintermedius* [76], was not detected in any of the analyzed *S. delphini* genomes. The presence of CRISPR/Cas and the type of *cas* genes was not related to the animal source of the isolation, thus the loci could have been acquired independently. *Staphylococcus aureus* pathogenicity islands (SaPI) are well described representatives of PICI and contribute to horizontal gene transfer [77]. In contrast to SaPI, *S. delphini* PICI SdPICI-1 was only observed in strains isolated from penguins and contained genes for phage-related head structural proteins and both terminase subunits, which has been observed in some PICIs of Gram-negative bacteria [78]. The finding of genomic island harboring leukocidin encoding genes *lukFS-I* among all analyzed strains confirms its ancient origin [25] and also its preservation among *S. delphini* strains in the polar environment. Virulence potential of all analyzed *S. delphini* strains is enhanced by the presence of SEC encoding genes, which have been previously detected in *S. pseudintermedius* isolates and associated with canine pyoderma [79,80]. Distribution of other toxin genes is variable among the strains (Table S3). Putative exfoliative toxin from P6456 strain has 53% amino acid identity with *S. pseudintermedius* exfoliative toxins EXI and ExpB [81,82]. Furthermore, an island with multiple paralog genes encoding trypsin-like serine proteases related to exfoliative toxins [83] was found in strain P5747 (Figure 5 and Table S3), being a possible virulence factor. 

*Staphylococcus delphini* genomes of the type strain, the strains representing two different phylogenetic branches of equine origin and the selected genomes of SIG isolates from other animals, were used for the comparative genomic analysis with focus on virulence, cell wall anchored proteins, and extracellular proteins (Table S3). These proteins most likely play a role in adaptation to the host and in a defence against host immunity. Homologs of *S. delphini* surface proteins were found in other SIG species, but many proteins were strain-specific, suggesting specific adaptation and virulence. However, there was no distinctive pattern of surface proteins corresponding to individual animal hosts. The location of these genes for surface proteins was predominantly on the chromosomes and not on the mobile genetic elements indicating both common ancestry and differential gene loss among strains as well as gene gain via horizontal transfer events. Despite numerous putative virulence factors found in analyzed genomes of *S. delphini*, further studies are needed to consider their role in animal pathogenesis or the possible risk of zoonotic infection to humans.

## 5. Emended Description of *Staphylococcus delphini* (Varaldo et al., 1988)

The description is based on the original description and observations of six isolates from penguins and eight reference strains from animals and human (Table 1). The morphological, biochemical, and physiological characteristics are generally those of the *S. delphini* description [15] with the exception of gelatin and lecithin hydrolysis and trehalose acidification, which were strain dependent. In contrast to type strain CCM 4115^T^ and reference strain CCM 4184, both from dolphins [15], all the studied *S. delphini* strains were gelatinase positive, but lecithinase negative and produced acid from trehalose. Below are the mentioned emended positive and negative test results not included in the original description.

Acid is produced from glycerol, ribose, galactose, and N-acetyl-glucosamine (API 50 CH). Esterase (C4), esterase-lipase (C8), acid phosphatase, pyrrolidonyl arylamidase, leucine arylamidase, and β-galactosidase positive (API ZYM). Resistant to bacitracin (0.04 U) and susceptible to furazolidone (100 µg). Negative oxidase, ornithine decarboxylase, esculin and starch hydrolysis, and growth at 20 °C. Acid production is negative from erythritol, D-arabinose, D-xylose, adonitol, β-methyl-D-xyloside, sorbose, rhamnose, dulcitol, inositol, sorbitol, α-methyl-D-mannoside, α-methyl-D-glucoside, amygdalin, arbutin, salicine, cellobiose, melibiose, inulin, melezitose, raffinose, starch, glycogen, D-lyxose, D-tagatose, D-fucose, L-fucose, D-arabitol, L-arabitol, gluconate, 2-keto-gluconate, and 5-keto-gluconate (API 50 CH). No enzymatic activity found for lipase (C14), valine arylamidase, cystine arylamidase, trypsin, α-chymotrypsin, α-galactosidase, β-glucuronidase, α-glucosidase, β-glucosidase, N-acetyl-β-glucosaminidase, and α-mannosidase (API ZYM). Variable reactions are shown in Table 1. Isolated from mucous membranes of different animals, occasionally humans. G + C content of 38 mol% calculated from whole genomic sequence.

## Figures and Tables

**Table 1:** Variable phenotypic reactions of *Staphylococcus intermedius* group strains under study.

Species	Strain	Source	Locality	Conventional Test Results								API 50 CH									API ZYM	
				COA	ARG	URE	TWE	GEL	Cas	E-Y	C45	GLY	MAN	SOR	ARB	MDG	LAC	TRE	GEN	TUR	N-PH	α-FU
*S. delphini*	CCM 4115^T^	dolphin	Italy	+	+	+	-	-	-	+	+	w	+	-	-	-	+	-	-	w	-	-
*S. delphini*	CCM 4184	dolphin	Italy	+	w	+	-	-	-	+	+	w	+	-	-	-	+	-	-	w	-	-
*S. delphini*	P5747	penguin	Antarctica	w	-	-	w	+	+	-	+	-	w	-	-	-	-	+	-	w	-	-
*S. delphini*	P5749	penguin	Antarctica	w	w	+	+	+	+	-	+	w	w	-	-	-	+	+	-	w	-	-
*S. delphini*	P5833	penguin	Antarctica	w	-	+	+	+	+	-	+	w	+	-	-	-	+	+	-	w	-	-
*S. delphini*	P5835	penguin	Antarctica	-	-	+	+	+	+	-	+	w	+	-	-	-	+	+	-	w	-	-
*S. delphini*	P6070	penguin	Antarctica	-	-	-	+	+	+	-	+	-	+	-	-	-	+	+	-	w	-	-
*S. delphini*	P6456	penguin	Antarctica	w	-	+	-	+	+	-	w	w	-	-	-	-	w	+	-	-	-	-
*S. delphini*	P12456	camel	France	w	w	+	-	+	+	-	-	+	+	-	w	-	+	+	+	w	+	-
*S. delphini*	P12457	horse	UK	+	w	+	-	+	+	-	-	w	+	+	-	-	w	+	+	w	w	w
*S. delphini*	P12458	horse	UK	w	w	+	+	+	+	-	+	w	w	-	-	-	+	+	-	-	w	-
*S. delphini*	CCM 2618	mink	Czechia	+	-	+	+	+	+	-	+	+	+	-	-	-	+	+	-	w	-	w
*S. delphini*	P12548	mink	Denmark	w	-	+	-	+	+	-	+	w	-	-	-	-	+	+	-	w	-	w
*S. delphini*	P12549	mink	Denmark	w	-	+	+	+	+	+	+	w	+	-	-	-	+	+	-	w	-	-
*S. delphini*	P12550	mink	Denmark	w	w	+	+	+	+	-	+	w	+	-	-	-	+	+	-	w	-	w
*S. delphini*	CCM 8998	human	USA	+	+	+	+	+	-	-	+	w	+	-	-	-	+	+	-	+	-	-
*S. pseudintermedius*	CCM 7315^T^	cat	Belgium	+	+	+	-	+	-	-	+	+	+	-	-	w	+	+	-	+	-	-
*S. pseudintermedius*	P7945	seal	Antarctica	w	w	+	-	+	+	-	-	w	-	-	-	-	+	+	-	-	-	-
*S. pseudintermedius*	P8480	seal	Antarctica	+	-	+	-	+	+	-	+	+	-	-	-	w	+	+	-	w	-	-
*S. pseudintermedius*	P8688	seal	Antarctica	+	-	+	-	+	+	-	-	w	-	-	-	-	+	+	-	-	w	-
*S. pseudintermedius*	P8720	seal	Antarctica	w	+	+	-	+	+	-	-	w	-	-	-	-	+	+	-	-	-	-
*S. pseudintermedius*	P8807	seal	Antarctica	+	+	+	-	+	+	-	w	+	-	-	-	w	+	+	-	w	-	-
*S. pseudintermedius*	P9111	seal	Antarctica	+	w	+	-	+	+	-	w	+	+	-	-	w	+	+	-	w	-	-
*S. pseudintermedius*	P12459	seal	Antarctica	w	-	+	-	+	+	-	+	w	-	-	-	w	+	+	-	-	-	-
*S. pseudintermedius*	P12460	seal	Antarctica	-	w	+	-	+	+	-	+	w	+	-	-	w	+	+	-	+	-	-
*S. pseudintermedius*	P12461	seal	Antarctica	w	w	+	-	+	+	-	+	+	-	-	-	w	+	+	-	+	-	-
*S. pseudintermedius*	P12462	seal	Antarctica	-	-	+	-	+	+	-	+	+	+	-	-	w	+	+	-	+	-	-
*S. pseudintermedius*	P12463	seal	Antarctica	-	w	+	-	+	+	-	+	+	-	-	-	w	+	+	-	+	-	-
*S. pseudintermedius*	P12464	seal	Antarctica	-	w	+	-	+	+	-	+	+	+	-	-	w	+	+	-	+	-	-
*S. pseudintermedius*	P12465	seal	Antarctica	w	w	+	-	+	+	-	+	+	-	-	-	-	+	+	-	+	-	-
*S. pseudintermedius*	P12466	seal	Antarctica	w	w	+	-	+	+	-	w	+	+	-	-	w	+	+	-	+	-	-
*S. pseudintermedius*	P12467	seal	Antarctica	+	w	+	-	+	+	-	+	+	-	-	-	w	+	+	-	-	-	-
*S. pseudintermedius*	P10574	seal	Antarctica	-	+	+	-	+	+	-	-	w	-	-	-	-	+	+	-	-	-	-
*S. intermedius*	CCM 5739^T^	pigeon	Czechia	w	-	+	-	+	+	-	+	w	+	-	-	w	+	+	+	w	-	-
*S. cornubiensis*	CCM 8997^T^	human	UK	+	+	+	+	+	+	+	+	+	+	-	-	-	+	+	-	+	-	-

**Legend:** COA, coagulase; ARG, arginine dihydrolase; URE, urease; TWE, hydrolysis of Tween 80; GEL, hydrolysis of gelatin; Cas, hydrolysis of casein; E-Y, egg-yolk reaction (lecithinase); C45, growth at 45 °C; GLY, acid from glycerol; MAN, acid from mannitol; SOR, acid from sorbitol; ARB, acid from arbutin; MDG, acid from α-methyl-D-glucoside; LAC, acid from lactose; TRE, acid from trehalose; GEN, acid from β-gentiobiose; TUR, acid from turanose; N-PH, naphthol-AS-BI-phosphohydrolase; α-FU, α-fucosidase; +, positive; -, negative; w, weak.

**Table 2:** Proposal of phenotypic traits suitable for differentiation of *Staphylococcus intermedius* group species.

Species	Strains	TWE	E-Y	MAN	MDG
*S. delphini*	16 strains	d (63%)	- (6%)	+ (88%)	- (0%)
*S. pseudintermedius*	17 strains	- (0%)	- (0%)	(-) (29%)	(+) (71%)
*S. intermedius*	CCM 5739^T^	-	-	+	w
*S. cornubiensis*	CCM 8997^T^	+	+	+	-

**Legend:** TWE, hydrolysis of Tween 80; E-Y, egg-yolk reaction (lecithinase); MAN, acid from mannitol; MDG, acid from α-methyl-D-glucoside; +, 85% to 100%; (+), 70% to 84%; d, 31% to 69%; (-), 15% to 29%; -, 0% to 14%; w, weak.

**Table 3:** Intergenomic distances between the genomes of *Staphylococcus intermedius* group strains in percent, represented by average nucleotide identity (ANI) and digitally derived genome-to-genome distances (GGD) emulating DNA–DNA hybridization values.

Strain	P5747		P6456		NCTC 12225^T^		215100905101-2		8086		NCTC 11048^T^		LMG 22219^T^		NW1^T^	
	GGD	ANI	GGD	ANI	GGD	ANI	GGD	ANI	GGD	ANI	GGD	ANI	GGD	ANI	GGD	ANI
*S. delphini* P5747	-	-	70.2	96.3	70.5	96.5	77.4	97.6	85.6	98.4	35.8	88.6	55.1	93.9	33.5	87.4
*S. delphini* P6456	70.2	96.3	-	-	87.5	98.7	69.5	96.3	69.0	96.3	35.5	88.4	51.3	93.1	33.2	87.2
*S. delphini* NCTC 12225^T^	70.5	96.5	87.5	98.7	-	-	70.4	96.6	70.1	96.4	35.9	88.6	51.2	93.1	33.5	87.4
*S. delphini* 215100905101-2	77.4	97.6	69.5	96.3	70.4	96.6	-	-	78.4	97.6	35.7	88.6	54.7	93.7	33.6	87.8
*S. delphini* 8086	85.6	98.4	69.0	96.3	70.1	96.4	78.4	97.6	-	-	35.6	88.6	54.9	93.9	33.6	87.5
*S. intermedius* NCTC 11048^T^	35.8	88.6	35.5	88.4	35.9	88.6	35.7	88.6	35.6	88.7	-	-	34.9	88.0	36.2	88.6
*S. pseudintermedius* LMG 22219^T^	55.1	93.9	51.3	93.1	51.2	93.1	54.7	93.7	54.9	93.9	34.9	88.0	-	-	32.9	87.2
*S. cornubiensis* NW1^T^	33.5	87.4	33.2	87.2	33.5	87.4	33.6	87.8	33.6	87.6	36.2	88.6	32.9	87.2	-	-

## Supplementary Materials

The following are available online at https://www.mdpi.com/2076-2607/8/2/204/s1: Table S1: *Staphylococcus* spp. strains identified in animal-related samples from James Ross Island and Seymour Island, Antarctica, Table S2: Comparison of selected genomic features of *Staphylococcus delphini* strains P5747 and P6456 from penguins, and *S. delphini* strains from other hosts, Table S3: Distribution of virulence factors, surface and extracellular proteins among the *Staphylococcus delphini* strains from different hosts and from the reference and type strains of other SIG species.
