# Supplementary material for: Characterization of Staphylococcus intermedius Group Isolates Associated with Animals from Antarctica and Emended Description of Staphylococcus delphini
Source: Microorganisms. 2020 Feb 1;8(2):204. doi: 10.3390/microorganisms8020204 (PMC7074773; doi:10.3390/microorganisms8020204)
Supplement: Supplementary file 1 [file microorganisms-08-00204-s001.pdf]

microorganisms

MDPI

Supplementary materials

Characterization of *Staphylococcus intermedius* Group Isolates Associated with Animals from Antarctica and Emended Description of *Staphylococcus delphini*

Veronika Vrbovská [1], Ivo Sedláček [2], Michal Zeman [1,2], Pavel Švec [2], Vojtěch Kovařovic [1], Ondrej Šedo [3], Monika Laichmanová [2], Jiří Doškař [1] and Roman Pantůček [1,*]

[1] Division of Genetics and Molecular Biology, Department of Experimental Biology, Faculty of Science, Masaryk University, Kotlářská 2, 611 37 Brno, Czech Republic;

[2] Czech Collection of Microorganisms, Department of Experimental Biology, Faculty of Science, Masaryk University, Kamenice 5, 625 00 Brno, Czech Republic;

[3] Central European Institute of Technology, Masaryk University, Kamenice 5, 625 00 Brno, Czech Republic;

[*] Correspondence: pantucek[at]sci.muni.cz; Tel.: +420-549-49-6379 (R.P.)

This document contains supplementary materials:

Table S1. *Staphylococcus* spp. strains identified in animal-related samples from James Ross Island and Seymour Island, Antarctica.
Table S2. Comparison of selected genomic features of *Staphylococcus delphini* strains P5747 and P6456 from penguins, and *S. delphini* strains from other hosts.
Table S3. Distribution of virulence factors, surface and extracellular proteins among the *Staphylococcus delphini* strains from different hosts and from the reference and type strains of other SIG species.

Table S1. *Staphylococcus* spp. strains identified in animal-related samples from James Ross Island and Seymour Island, Antarctica.

Strain No.	Source	Year of isolation	Closest match	Partial 16S rRNA gene - identification score
***Staphylococcus aureus - Staphylococcus epidermidis* phylogenetic clade**				
P5738	penguin, beak	2014	*Staphylococcus aureus/S. argenteus/S. schweitzeri*	100%
P5744	penguin, cloaca	2014	*Staphylococcus aureus/S. argenteus/S. schweitzeri*	100%
P5772	skua bird droppings	2014	*Staphylococcus aureus/S. argenteus/S. schweitzeri*	100%
P7183	penguin, beak	2016	*Staphylococcus aureus/S. argenteus/S. schweitzeri*	100%
P7781	penguin, beak	2017	*Staphylococcus aureus/S. argenteus/S. schweitzeri*	100%
P7797	seal, mouth	2017	*Staphylococcus aureus/S. argenteus/S. schweitzeri*	100%
P8154	seal, mouth	2017	*Staphylococcus aureus/S. argenteus/S. schweitzeri*	100%
P8452	lake water	2017	*Staphylococcus aureus/S. argenteus/S. schweitzeri*	100%
P8750	seal, anus	2017	*Staphylococcus aureus/S. argenteus/S. schweitzeri*	100%
P8753	seal, anus	2017	*Staphylococcus aureus/S. argenteus/S. schweitzeri*	100%
P8992	seal, anus	2017	*Staphylococcus aureus/S. argenteus/S. schweitzeri*	100%
P9166	seal, mouth	2017	*Staphylococcus aureus/S. argenteus/S. schweitzeri*	100%
P9115	seal, mouth	2017	*Staphylococcus aureus/S. argenteus/S. schweitzeri*	99.61%
R 6/2	elephant seal, anus	2019	*Staphylococcus aureus/S. argenteus/S. schweitzeri*	100%
P4768	unknown, droppings	2013	*Staphylococcus epidermidis*	100%
P4961	unknown, feather	2013	*Staphylococcus epidermidis*	100%
P8092	seal, anus	2017	*Staphylococcus epidermidis*	100%
P8579	seal, mouth	2017	*Staphylococcus epidermidis*	100%
P8586	seal, anus	2017	*Staphylococcus epidermidis*	100%
P8725	seal, anus	2017	*Staphylococcus epidermidis*	100%
P8816	skua, droppings	2017	*Staphylococcus epidermidis*	100%
P9120	elephant seal, mouth	2017	*Staphylococcus epidermidis*	99%
P9131	seal, droppings	2017	*Staphylococcus epidermidis*	99.61%
P9151	seal, anus	2017	*Staphylococcus epidermidis*	100%
P10571	seal, anus	2018	*Staphylococcus epidermidis*	99.42%
R 5/2	elephant seal, anus	2019	*Staphylococcus epidermidis*	100%
T 8/2	seal, anus	2019	*Staphylococcus epidermidis*	100%
T 10/7	seal, mouth	2019	*Staphylococcus epidermidis*	100%
T 11/2	seal, mouth	2019	*Staphylococcus epidermidis*	100%
T 18/4	seal, mouth	2019	*Staphylococcus epidermidis*	100%
T 19/5	seal, mouth	2019	*Staphylococcus epidermidis*	100%
T 19/6	seal, mouth	2019	*Staphylococcus epidermidis*	100%
T 49/3	seal, anus	2019	*Staphylococcus epidermidis*	99.43%
T 57/4	seal, mouth	2019	*Staphylococcus epidermidis*	99.43%
T 62/2	seal, anus	2019	*Staphylococcus epidermidis*	99.81%
T 66/2	seal, anus	2019	*Staphylococcus epidermidis*	99.81%
P8539	seal, mouth	2017	*Staphylococcus caprae/S. capitis*	100%
P8707	seal, anus	2017	*Staphylococcus caprae/S. capitis*	100%
P8806	seal, anus	2017	*Staphylococcus caprae/S. capitis*	100%
P8859	seal, mouth	2017	*Staphylococcus caprae/S. capitis*	100%
P8863	seal, anus	2017	*Staphylococcus caprae/S. capitis*	100%
P9104	seal, mouth	2017	*Staphylococcus caprae/S. capitis*	99.61%
P9107	seal, mouth	2017	*Staphylococcus caprae/S. capitis*	100%
P9145	seal, mouth	2017	*Staphylococcus caprae/S. capitis*	100%
P9190	seal, anus	2017	*Staphylococcus caprae/S. capitis*	100%
T 28/1	seal, mouth	2019	*Staphylococcus caprae/S. capitis*	100%
T 36/2	seal, anus	2019	*Staphylococcus caprae/S. capitis*	99.62%
T 38/2	seal, anus	2019	*Staphylococcus caprae/S. capitis*	99.23%
P7134	penguin, droppings	2016	*Staphylococcus warneri*	100%
P7141	unknown, droppings	2016	*Staphylococcus warneri*	99.81%
P8643	penguin, beak	2017	*Staphylococcus warneri*	100%
P10576	seal, anus	2018	*Staphylococcus warneri*	99.81%
T 17/4	seal, anus	2019	*Staphylococcus pasteuri*	100%
T 10/8	seal, mouth	2019	*Staphylococcus pasteuri*	100%

Table S1. Continued.

Strain No.	Source	Year of isolation	Closest match	Partial 16S rRNA gene - identification score
***Staphylococcus haemolyticus* phylogenetic clade**				
P5756	fresh skua bird droppings	2014	*Staphylococcus haemolyticus*	100%
P5757	fresh skua bird droppings	2014	*Staphylococcus haemolyticus*	99.61%
P6353	penguin, cloaca	2015	*Staphylococcus haemolyticus*	100%
P7139	unknown, droppings	2016	*Staphylococcus haemolyticus*	99.61%
P7142	skua, droppings	2016	*Staphylococcus haemolyticus*	100%
P7167	skua, droppings	2016	*Staphylococcus haemolyticus*	100%
P8018	seal, anus	2017	*Staphylococcus haemolyticus*	100%
P8019	seal, anus	2017	*Staphylococcus haemolyticus*	100%
P8184	seal, mouth	2017	*Staphylococcus haemolyticus*	100%
P8567	seal, anus	2017	*Staphylococcus haemolyticus*	99.81%
P8656	seal, droppings	2017	*Staphylococcus haemolyticus*	100%
P8696	seal, anus	2017	*Staphylococcus haemolyticus*	99.61%
P8721	seal, mouth	2017	*Staphylococcus haemolyticus*	100%
P8782	seal, anus	2017	*Staphylococcus haemolyticus*	100%
P9002	seal, mouth	2017	*Staphylococcus haemolyticus*	99.61%
P9097	seal, anus	2017	*Staphylococcus haemolyticus*	99.61%
P9130	skua, droppings	2017	*Staphylococcus haemolyticus*	99.61%
P9139	seal, mouth	2017	*Staphylococcus haemolyticus*	100%
P9140	seal, mouth	2017	*Staphylococcus haemolyticus*	99.61%
P9175	penguin, cloaca	2017	*Staphylococcus haemolyticus*	99.61%
P9191	seal, mouth	2017	*Staphylococcus haemolyticus*	100%
P10074	seal, anus	2018	*Staphylococcus haemolyticus*	99.62%
F 1/5	skua, droppings	2019	*Staphylococcus haemolyticus*	100%
T 14/1	seal, mouth	2019	*Staphylococcus haemolyticus*	100%
T 14/3	seal, mouth	2019	*Staphylococcus haemolyticus*	99.62%
T 43/1	seal, mouth	2019	*Staphylococcus haemolyticus*	99.04%
T 47/4	seal, anus	2019	*Staphylococcus haemolyticus*	99.04%
T 51/1	seal, anus	2019	*Staphylococcus haemolyticus*	99.62%
P9148	seal, mouth	2017	*Staphylococcus hominis*	99.22%
***Staphylococcus simulans* phylogenetic clade**				
P9179	penguin, droppings	2017	*Staphylococcus auricularis*	100%
***Staphylococcus saprophyticus* phylogenetic clade**				
P7145	skua, droppings	2016	*Staphylococcus saprophyticus /S. eadaphicus*	99.8%
P7160	penguin, cloaca	2016	*Staphylococcus saprophyticus /S. eadaphicus*	100%
T 54/8A	seal, mouth	2019	*Staphylococcus saprophyticus /S. eadaphicus*	100%
T 60/3	seal, anus	2019	*Staphylococcus saprophyticus /S. eadaphicus*	99.81%
P8769	seal, mouth	2017	*Staphylococcus saprophyticus /S. eadaphicus*	100%
P8490	seal, anus	2017	*Staphylococcus saprophyticus /S. eadaphicus*	100%
T 52/5	seal, mouth	2019	*Staphylococcus saprophyticus /S. eadaphicus*	99.42%
T 54/3	seal, anus	2019	*Staphylococcus saprophyticus /S. eadaphicus*	99.42%
T 69/4	seal, mouth	2019	*Staphylococcus saprophyticus /S. eadaphicus*	99.61%
T 40/4	seal, anus	2019	*Staphylococcus cohnii*	99.62%
T 54/5	seal, mouth	2019	*Staphylococcus cohnii*	99.62%
T 56/2	seal, anus	2019	*Staphylococcus cohnii*	99.81%
T 56/3A	seal, anus	2019	*Staphylococcus cohnii*	99.81%
P10515	seal, mouth	2018	*Staphylococcus cohnii*	98.08%
P7715	penguin, droppings	2017	*Staphylococcus equorum*	99.81%
T 51/3	seal, anus	2019	*Staphylococcus equorum*	99.62%
T 54/7	seal, mouth	2019	*Staphylococcus succinus*	99.42%
T 61/2	seal, anus	2019	*Staphylococcus succinus*	99.62%

Table S1. Continued.

Strain No.	Source	Year of isolation	Closest match	Partial 16S rRNA gene - identification score
Staphylococcus hyicus - Staphylococcus intermedius **phylogenetic clade**				
P5747	penguin, beak	2014	*Staphylococcus intermedius* group	100%
P5749	penguin, cloaca	2014	*Staphylococcus intermedius* group	100%
P5833	penguin, beak	2014	*Staphylococcus intermedius* group	100%
P5835	penguin, cloaca	2014	*Staphylococcus intermedius* group	100%
P6070	penguin, beak	2014	*Staphylococcus intermedius* group	100%
P6456	penguin, beak	2015	*Staphylococcus intermedius* group	100%
P7945	seal, anus	2017	*Staphylococcus intermedius* group	100%
P8480	seal, anus	2017	*Staphylococcus intermedius* group	100%
P8688	seal, anus	2017	*Staphylococcus intermedius* group	100%
P8720	seal, mouth	2017	*Staphylococcus intermedius* group	100%
P8807	seal, anus	2017	*Staphylococcus intermedius* group	100%
P9111	seal, mouth	2017	*Staphylococcus intermedius* group	100%
P10574	seal, anus	2018	*Staphylococcus intermedius* group	100%
P12459	seal, anus	2019	*Staphylococcus intermedius* group	100%
P12460	seal, anus	2019	*Staphylococcus intermedius* group	100%
P12461	seal, mouth	2019	*Staphylococcus intermedius* group	100%
P12462	seal, anus	2019	*Staphylococcus intermedius* group	100%
P12463	seal, anus	2019	*Staphylococcus intermedius* group	100%
P12464	seal, mouth	2019	*Staphylococcus intermedius* group	100%
P12465	seal, anus	2019	*Staphylococcus intermedius* group	100%
P12466	seal, anus	2019	*Staphylococcus intermedius* group	100%
P12467	seal, anus	2019	*Staphylococcus intermedius* group	100%
P5746	penguin, cloaca	2014	*Staphylococcus schleiferi*	100%
P5841	penguin, beak	2014	*Staphylococcus schleiferi*	100%
P6543	seal, anus	2015	*Staphylococcus schleiferi*	100%
P8578	seal, anus	2017	*Staphylococcus schleiferi*	100%
R 9/1	elephant seal, anus	2019	*Staphylococcus schleiferi*	99.81%
F 2/1	skua, droppings	2019	*Staphylococcus schleiferi*	100%
Staphylococcus sciuri **phylogenetic clade**				
P4774	unknown, droppings	2013	*Staphylococcus sciuri*	100%
P5740	penguin, beak	2014	*Staphylococcus sciuri*	100%
P5742	penguin, cloaca	2014	*Staphylococcus sciuri*	100%
P5748	penguin, beak	2014	*Staphylococcus sciuri*	100%
P5761	fresh kelp gull droppings	2014	*Staphylococcus sciuri*	100%
P5762	fresh kelp gull droppings	2014	*Staphylococcus sciuri*	100%
P5768	fresh skua bird droppings	2014	*Staphylococcus sciuri*	100%
P5844	penguin, beak	2014	*Staphylococcus sciuri*	100%
P5846	penguin, cloaca	2014	*Staphylococcus sciuri*	100%
P6153	penguin, beak	2014	*Staphylococcus sciuri*	100%
P6183	fresh skua bird droppings	2014	*Staphylococcus sciuri*	100%
P6454	penguin, beak	2015	*Staphylococcus sciuri*	100%
P6464	penguin, beak	2015	*Staphylococcus sciuri*	100%
P7149	kelp gull, droppings	2016	*Staphylococcus sciuri*	99.81%
P9174	penguin, beak	2017	*Staphylococcus sciuri*	99.81%
R 3/5	elephant seal, anus	2019	*Staphylococcus sciuri*	99.43%
R 4/3	elephant seal, mouth	2019	*Staphylococcus sciuri*	99.43%
R 4/7	elephant seal, mouth	2019	*Staphylococcus sciuri*	99.43%
P5770	penguin, droppings	2014	*Staphylococcus sciuri/S.fleuretti/S. vitulinus*	99.02%
F 5/4	unknown, droppings	2019	*Staphylococcus sciuri/S.fleuretti/S. vitulinus*	99.02%

Table S2. Comparison of selected genomic features of *Staphylococcus delphini* strains P5747 and P6456 from penguins, and *S. delphini* strains from other hosts.

Genome		*S. delphini* P5747 (penguin)	*S. delphini* P6456 (penguin)	*S. delphini* NCTC 12225^{T} (dolphin)	*S. delphini* 8086 (horse)	*S. delphini* 215100905101-2 (horse)
WGS Project no.		WNLD00000000.1	WNLE00000000.1	LR134263.1	CAIA00000000.1	MWUT00000000.1
Size (Mb)		2.54	2.65	2.80	2.51	2.53
Contigs		47	104	1	211	30
GC content (mol%)		38.2	38.1	37.8	38.3	38.3
Total genes		2488	2572	2633	2399	2394
Protein coding sequences		2292	2386	2452	2315	2281
Genes with clusters of orthologous groups[a]		2179	2260	2201	2214	2220
Prophages		P5747-1 (41.2 kb); P5747-2 (44.4 kb)	P6456-1*, P6456-2*	SPβ-like 12225 (120.3 kb)	8086-1*, 8086-2*	215100905101-2-1 (39.5 kb)
Plasmids[b]		pSD1 (3.2 kb); pSD2 (3.1 kb); pSD3 (2.5 kb)	pSD2 (3.1 kb); pSD3 (2.5 kb); pSD4 (3.1 kb)[c]	-	-	-
Phage-inducible chromosomal islands		SdPICI-1 (13.7 kb)	SdPICI-1 (13.7 kb)	-	-	-
Other chromosomal island		SdCI$_{lukSF\text{-}I,\ ula}$ (11.5 kb), SdCI$_{SEC}$ (9.5 kb), SdCI$_{Serine_protease}$ (7.3 kb)	SdCI$_{lukSF\text{-}I,\ ula}$ (11.5 kb)	SdCI$_{lukSF\text{-}I,\ ula}$ (11.5 kb), SdCI$_{12225}$ (14.8 kb)	SdCI$_{lukSF\text{-}I,\ ula}$ (11.5 kb), SdCI$_{Serine_protease}$ (12.1 kb)[d]	SdCI$_{lukSF\text{-}I,\ ula}$ (11.5 kb)
Subcelluar localization of encoded proteins[e]	Cytoplasmic	1350	1384	1418	1332	1303
	Cytoplasmic Membrane	650	671	670	634	630
	Cellwall	34	34	31	39	33
	Extracellular	54	46	47	42	38
	Unknown	204	251	286	268	277
RM systems		type I (*hsdMSR*), type II (HindIII)	type I (incomplete - *hsdMS*), type II (HindIII)	type I (*hsdMSR*), type II (FokI)	type I (*hsdMSR*)	type I (*hsdMSR*)
CRISPR-Cas		type III-A	-	type II-C; type III-A	-	type II-C

* phages are incomplete and dispersed on multiple contigs

[a] core genome consist of 1899 structural genes in given set

[b] all detected plasmids are cryptic, encoding Rep protein and hypothetical proteins

[c] plasmid sequences on multiple contigs, unable to distinguish between one or more separated plasmids

[d] SdCI$_{Serine_protease}$ is on two separate contigs for strain 8086, the length is approximate

[e] predicted by PSORTb 3.0

Table S3. Distribution of virulence factors, surface and extracellular proteins among the *Staphylococcus delphini* strains from different hosts and from the reference and type strains of other SIG species.

* frameshifted and marked as a pseudogene, locus tag is indicated instead protein id.

Class	Predicted function	Gene	Genome / Protein accession numbers											
			S. delphini NCTC 12225^T (dolphin)	*S. delphini* P5747 (penguin)	*S. delphini* P6456 (penguin)	*S. delphini* 215100905101-2 (horse)	*S. delphini* 8086 (horse)	*S. delphini* 14S03313-1 (horse)	*S. delphini* 14S03318-1 (marten)	*S. delphini* 14S03309-1 (pigeon)	*S. pseudintermedius* LMG 22219^T (cat)	*S. pseudintermedius* P8688 (seal)	*S. intermedius* NCTC 11048^T (pigeon)	*S. cornubiensis* NW1^T (human)
			GCF_900636325.1	GCF_009720305.1	GCF_009720295.1	GCF_002369695.1	GCF_000308115.1	GCF_002374125.1	GCF_002369645.1	GCF_002374115.1	GCF_001792775.2	GCA_009939245.1	GCF_900458545.1	GCF_900183575.1
Toxins	Beta-hemolysin/ Sphingomyelin phosphodiesterase C	*sph*	-	WP_155260711.1	-	-	WP_019165692.1	-	WP_096547225.1	-	-	-	-	WP_086428551.1
		sph	WP_096540432.1	WP_155262035.1	WP_155259174.1	WP_096540432.1	WP_019166636.1	WP_096606037.1	WP_096544224.1	WP_096555042.1	WP_014614737.1	NCJ14254.1	SUM47755.1	WP_086428472.1
	Enterotoxin A	*sea*	-	-	-	-	-	-	-	-	WP_096536596.1	-	-	-
	Enterotoxin C	*sec*	WP_096596479.1	WP_155261879.1	WP_155260216.1	WP_096542243.1	WP_019166892.1	WP_096542243.1	WP_096543689.1	WP_096662110.1	WP_065354460.1	NCJ15066.1	SUM47431.1	WP_086429337.1
	Exotoxin/superantigen	*set15*	WP_096598118.1	WP_155260627.1	WP_155260428.1	WP_096539625.1	WP_019166911.1	WP_096604705.1	WP_096546265.1	WP_096591578.1	WP_103263609.1	NCJ15602.1	SUM45315.1	WP_086428748.1
		set26	-	-	-	-	-	-	WP_096546200.1	-	-	-	-	-
		-	-	-	-	-	-	-	-	-	-	-	SUM47717.1	-
	Exfoliative toxin	*eta* -like	-	-	-	-	-	-	-	-	WP_014614890.1	NCJ15629.1	-	-
		etb -like	-	-	WP_019165050.1	-	-	WP_096604575.1	WP_096546202.1	-	-	-	-	-
		etd -like	-	FMF08_11915*	-	-	-	-	-	-	-	-	-	-
		-	-	-	-	-	-	-	-	-	WP_070407803.1	-	-	-
	Leukotoxin	*lukF-I*	WP_096598532.1	WP_096555970.1	WP_155259183.1	WP_096541993.1	WP_019165928.1	WP_096606640.1	WP_096544903.1	WP_096661737.1	WP_070407634.1	NCJ14038.1	SUM46393.1	WP_086427464.1
		lukS-I	WP_096598534.1	WP_155261268.1	WP_155259607.1	WP_096541995.1	WP_019165929.1	WP_096606638.1	WP_096544905.1	WP_096661738.1	WP_014613568.1	NCJ14037.1	SUM46394.1	WP_086427465.1
Virulence related enzymes	Staphylocoagulase precursor	*coa*	WP_096595977.1	WP_155260894.1	WP_155260427.1	WP_096539624.1	WP_019166910.1	WP_096604707.1	WP_096546267.1	WP_096591580.1	WP_103263608.1	NCJ15601.1	SUM45313.1	WP_086428747.1
	Lipase	*geh*	WP_096596065.1	WP_155260818.1	WP_155259218.1	WP_096595680.1	WP_019165093.1	WP_096604375.1	-	-	WP_015729748.1<br>WP_063279060.1	NCJ15175.1<br>NCJ15176.1<br>NCJ15626.1	SUM45626.1	WP_086429553.1
		lip	WP_096596422.1<br>WP_096596423.1	WP_155260932.1	WP_155259218.1	WP_096595746.1	WP_019166331.1	-	WP_096544825.1<br>WP_096544823.1	WP_096591144.1	WP_100006587.1<br>WP_014612949.1	GWD55_09205*	-	WP_086429052.1
	Alkaline phosphatase III	*phoA*	WP_096596377.1	WP_155260883.1	WP_155259820.1	WP_096541810.1	WP_019167069.1	WP_096605845.1	WP_096546872.1	WP_096593460.1	WP_103263622.1	NCJ15122.1	SUM45704.1	WP_086428916.1
	Hyaluronate lyase	*hysA*	-	WP_155261881.1	-	-	-	-	-	-	-	-	-	-
	Phospholipase C	*plc*	-	-	WP_155259517.1	-	-	-	-	-	-	-	-	-
	Sialidase B	*gndA*	-	WP_155261448.1	-	-	WP_019165693.1	-	WP_096547227.1	-	WP_014614355.1	GWD55_06485*	-	WP_086428552.1
	Serine protease	*splA*	WP_096598594.1<br>WP_096598715.1	WP_155261246.1	-	-	WP_019165413.1	WP_096606080.1	-	WP_096591685.1	WP_096535728.1	-	-	WP_086427425.1<br>WP_086427426.1
		sspA	-	-	-	-	-	-	-	-	-	-	-	WP_086429394.1
		-	-	WP_155260725.1	-	-	WP_019165718.1	-	-	WP_096591357.1	WP_037542545.1	-	SUM45481.1	WP_086428589.1
		-	-	-	-	-	-	-	-	-	-	GWD55_00725*	SUM45442.1	-
		-	-	WP_155262083.1<br>WP_155261999.1<br>WP_155262084.1<br>WP_155262000.1<br>WP_155262001.1<br>WP_155262002.1<br>WP_155262003.1	-	-	WP_019166273.1<br>WP_019166274.1<br>WP_019166275.1<br>WP_019166276.1<br>WP_019166277.1<br>WP_019166278.1<br>WP_019166279.1<br>WP_019166280.1<br>WP_019166281.1<br>WP_019166282.1<br>WP_019166283.1<br>WP_019166284.1<br>WP_019166285.1	-	-	-	-	-	-	-
	Superoxide dismutase	*sodA*	WP_096597017.1	WP_155261417.1	WP_155259760.1	WP_096541495.1	WP_019165219.1	WP_096547006.1	WP_096547006.1	WP_019165219.1	WP_014613806.1	NCJ15362.1	SUM46636.1	WP_086427684.1
	Zinc metalloproteinase aureolysin	*aur*	WP_096596453.1	WP_155260949.1	WP_155259240.1	WP_096542883.1	WP_019164961.1	WP_096606583.1	WP_096544762.1	WP_096591102.1	WP_103263571.1	NCJ15203.1	SUM45806.1	WP_086427981.1
	Thermonuclease	*nuc*	WP_096596341.1<br>WP_096598721.1	WP_155261540.1<br>WP_155261541.1	WP_096596341.1<br>WP_155259933.1	WP_096541348.1<br>WP_096540971.1	WP_019165402.1<br>WP_019165406.1	WP_096606537.1<br>WP_096606164.1	WP_096545353.1<br>WP_096545348.1	WP_096545353.1<br>WP_096591696.1	WP_015729076.1<br>WP_014614009.1	NCJ14857.1<br>NCJ14855.1	SUM46842.1<br>SUM46845.1	WP_086427865.1<br>WP_086427867.1
Iron uptake	Periplasmic binding protein-dependent ABC transport systems	*vtcC*	-	-	WP_155260174.1	WP_096589094.1	-	WP_096604809.1	-	-	-	-	-	-

Table S3. Continued.

Class	Predicted function	Gene	Genome / Protein accession numbers											
			S. delphini NCTC 12225^T (dolphin)	*S. delphini* P5747 (penguin)	*S. delphini* P6456 (penguin)	*S. delphini* 215100905101-2 (horse)	*S. delphini* 8086 (horse)	*S. delphini* 14S03313-1 (horse)	*S. delphini* 14S03318-1 (marten)	*S. delphini* 14S03309-1 (pigeon)	*S. pseudintermedius* LMG 22219^T (cat)	*S. pseudintermedius* P8688 (seal)	*S. intermedius* NCTC 11048^T (pigeon)	*S. cornubiensis* NW1^T (human)
			GCF_900636325.1	GCF_009720305.1	GCF_009720295.1	GCF_002369695.1	GCF_000308115.1	GCF_002374125.1	GCF_002369645.1	GCF_002374115.1	GCF_001792775.2	GCA_009939245.1	GCF_900458545.1	GCF_900183575.1
Immune evasion	Adenosine synthase	*adsA*	WP_096596233.1	WP_155260988.1	WP_155259296.1	WP_096589112.1	WP_019165402.1	WP_096606818.1	WP_096544639.1	-	WP_037542320.1	NCJ14595.1	SUM45865.1	WP_086428047.1
	Polysaccharide capsule	-	WP_096597982.1<br>WP_096597232.1<br>WP_096596211.1	WP_155260716.1<br>WP_155261758.1<br>WP_155261047.1<br>WP_155261153.1<br>WP_155260717.1	WP_155259479.1<br>WP_155260057.1<br>WP_096542578.1	WP_096543029.1<br>WP_096543007.1<br>WP_096542578.1	WP_019165802.1<br>WP_019167167.1<br>WP_026066994.1<br>WP_039838313.1	WP_096606426.1<br>WP_096605264.1<br>WP_096542578.1	WP_096546510.1<br>WP_096544497.1<br>WP_096544283.1	WP_019165802.1<br>WP_096592328.1<br>WP_096661961.1<br>WP_096591365.1	WP_014613151.1<br>WP_103263702.1<br>WP_014614306.1	NCJ13750.1<br>NCJ14507.1<br>NCJ13577.1	SUM45956.1<br>SUM45471.1<br>SUM46132.1<br>SUM47211.1<br>SUM45472.1	WP_086428572.1<br>WP_086428569.1<br>WP_086428134.1<br>WP_086429146.1<br>WP_086428571.1<br>WP_086428581.1<br>WP_086429480.1<br>WP_086428570.1
		galE	WP_096597290.1	WP_019166591.1	WP_155259394.1	WP_019166591.1	WP_019166591.1	WP_019166591.1	WP_096544436.1	WP_096590871.1	WP_015729527.1	NCJ14411.1	SUM45989.1	WP_086428165.1
	Immunodomiant antigen	*isaA*	WP_096596443.1	WP_155260946.1	WP_155259231.1	WP_096542905.1	WP_019164949.1	WP_096606599.1	WP_096544786.1	WP_019164949.1	WP_014612972.1	NCJ15198.1	SUM45794.1	WP_086427976.1
		isaB	WP_096598090.1	WP_155262118.1	WP_155260417.1	WP_096539601.1	WP_019166374.1	WP_096604729.1	WP_096546318.1	WP_096662084.1	WP_014614839.1	NCJ15585.1	SUM45288.1	WP_086428728.1
	Secretory antigen precursor	*ssaA*	WP_096597266.1	WP_155261055.1	WP_155259384.1	WP_096542552.1	WP_019165367.1	WP_096605640.1	WP_096544468.1	WP_096590891.1	WP_015729537.1	NCJ14491.1	SUM45972.1	WP_086428149.1
			WP_096597260.1	WP_155261054.1	WP_096597260.1	WP_096542560.1	WP_019165363.1	WP_096542560.1	WP_096544476.1	WP_019165363.1	WP_014613162.1	NCJ14495.1	SUM45968.1	WP_086428146.1
Adherence	Clumping factor A	*clfA*	-	WP_155260821.1<br>WP_155260820.1	WP_155259885.1<br>WP_155259118.1	WP_096543116.1	WP_014614797.1	-	WP_096546353.1<br>WP_096546065.1	WP_096662080.1	-	NCJ15232.1	SUM45308.1<br>SUM45629.1	-
	Clumping factor B	*clfB*	-	-	-	-	WP_019167000.1	-	-	-	-	-	-	-
	Collagen adhesion	*cna*	-	-	-	-	-	WP_096606518.1	WP_096546586.1	-	-	-	-	-
	Elastin binding protein	*ebpS*	WP_096596248.1	WP_019165998.1	WP_096542773.1	WP_096541026.1	WP_019165282.1	WP_096605307.1	WP_096545540.1	WP_096661794.1	WP_099987111.1	NCJ14989.1	SUM46715.1	WP_086427749.1
	Fibronectin binding proteins	*fnbB*	WP_096596309.1	WP_155261510.1	WP_155259848.1	WP_096541241.1	WP_019165356.1	WP_096606482.1	WP_096545417.1	WP_096661811.1	WP_112424790.1	-	SUM46794.1	WP_086427824.1
	Intercellular adhesin	*icaA*	WP_096596031.1	WP_155260651.1	WP_155259087.1	WP_096540014.1	WP_019166834.1	-	-	WP_019166834.1	WP_101431304.1	GWD55_09330*	SUM45578.1	WP_086428671.1
		icaB	WP_096596029.1	-	WP_155259341.1	WP_096540010.1	WP_019166832.1	WP_096604431.1	WP_096546001.1	WP_096662060.1	WP_103263494.1	NCJ15261.1	SUM45576.1	WP_086428669.1
		icaC	WP_096596028.1	WP_155260791.1	WP_155259085.1	WP_096540008.1	WP_019166831.1	WP_096604433.1	WP_096545999.1	WP_096593797.1	WP_014612782.1	NCJ15262.1	SUM45575.1	WP_086428668.1
	Ser-Asp rich fibrinogen-binding proteins	*sdrC*	WP_096596063.1	-	-	-	-	-	-	-	-	-	SUM43760.1<br>SUM43658.1	-
		sdrD	WP_096598315.1	WP_155262069.1	WP_155260135.1	WP_096595588.1	WP_026067054.1	WP_096606702.1	WP_096543518.1	WP_096661881.1	-	NCJ13608.1	SUM47308.1	WP_086429410.1
		sdrE	WP_096596064.1	WP_155260816.1	WP_155259116.1	WP_096595684.1<br>WP_158224546.1	WP_019165862.1	WP_096604377.1	WP_096546059.1	WP_096662054.1	-	-	SUM45624.1	WP_086428706.1<br>WP_119184415.1
	MSCRAMM family adhesin	-	-	-	-	-	-	-	WP_142302650.1	-	-	-	-	-
	IgG-binding protein	*sbi*	-	-	-	-	-	-	-	WP_096662037.1	-	-	-	-
	Staphylococcal protein A	*spa2*	-	-	-	-	-	-	-	WP_096591397.1	-	-	SUM45452.1	WP_086428550.1
	N-acetylmuramoyl-L-alanine amidase	*atl*	WP_096596071.1<br>WP_096595957.1	WP_155261763.1	WP_155260061.1<br>WP_155260554.1	WP_096595580.1<br>WP_096539908.1	WP_026066996.1<br>WP_019165733.1	WP_096605050.1<br>WP_096604517.1	WP_096543477.1<br>WP_096546602.1	WP_096592220.1<br>WP_096662014.1	WP_014614312.1<br>WP_014612723.1	NCJ13582.1<br>NCJ13896.1	SUM47217.1<br>SUM45489.1	WP_086429636.1<br>WP_086428606.1
Surface protein anchoring	Lipoprotein diacylglyceryl transferase	*lgt*	WP_096596548.1	WP_155261892.1	WP_155260237.1	WP_096542309.1	WP_019166113.1	WP_096605044.1	WP_014613931.1	WP_096593999.1	WP_014614497.1	NCJ15041.1	SUM47458.1	WP_086428514.1
	Lipoprotein-specific signal peptidase II	*lspA*	WP_096596174.1	WP_019165555.1	WP_155259968.1	WP_096540618.1	WP_019165555.1	WP_096540618.1	WP_096543842.1	WP_019165555.1	WP_014614161.1	NCJ14715.1	-	WP_086429113.1
	Cell wall anchor protein	*sasF*	WP_096596397.1	WP_155260907.1	WP_155259194.1	WP_096595748.1	WP_019166502.1	WP_096605801.1	WP_096544873.1	WP_096591196.1	WP_103263651.1	NCJ15154.1	SUM45746.1	WP_086429024.1
	Surface 5'-nucleotidase	*sasH*	-	-	-	-	WP_019165558.1	-	-	-	-	-	-	-
	YSIRK-type signal peptide-containing proteins with LPXTG domain	-	WP_126489868.1	WP_155260739.1<br>WP_155260895.1	WP_155259183.1	WP_096595750.1	WP_019165736.1<br>WP_019165898.1	WP_096605823.1	WP_096546834.1<br>WP_096546600.1	WP_096662013.1	WP_112424773.1<br>WP_142671672.1	NCJ13920.1<br>NCJ15309.1	SUM45730.1	WP_119184408.1<br>WP_119184405.1
		-	WP_096596001.1	WP_155260957.1	WP_155259643.1	WP_096539956.1	WP_019166800.1	WP_096604471.1	WP_096545946.1	WP_096662010.1	WP_103263484.1	GWD55_09480*<br>GWD55_09480*	-	WP_086428636.1
		-	WP_096598635.1	WP_155260642.1	WP_155260443.1	WP_096539671.1	WP_019166934.1	WP_096604680.1	WP_096546221.1	WP_096662072.1	WP_063279061.1	NCJ15627.1	SUM45341.1	WP_086428768.1
		-	WP_096596063.1	-	WP_155259115.1	-	-	-	-	-	-	-	-	-
	Unknown LPXTG domain containing proteins	-	-	WP_155261832.1	-	WP_096542755.1	WP_019165986.1	-	-	WP_096592157.1	WP_014614371.1	NCJ13643.1	SUM45443.1	WP_086428952.1
		-	-	-	WP_155260512.1	-	WP_019166773.1	-	WP_096546656.1	WP_096662018.1	-	-	SUM45461.1	WP_086428561.1
		-	-	WP_155261511.1	-	-	WP_019166538.1	-	-	-	-	NCJ14904.1	-	-
		-	-	WP_155260826.1	-	-	WP_083849007.1	-	-	-	WP_142671673.1	NCJ15230.1	-	WP_086429411.1
		-	-	-	-	-	-	-	-	-	-	-	SUM45492.1	-
		-	-	-	-	-	-	-	-	-	-	-	SUM45729.1	-
		-	-	-	-	WP_096595626.1	-	-	-	-	-	-	-	-
		-	-	-	-	-	WP_039838176.1	-	-	-	-	-	-	-
		-	-	-	-	-	WP_039838492.1	-	-	-	-	-	-	-
		-	-	-	WP_155259884.1	-	-	-	-	-	-	-	-	-
		-	-	-	-	-	WP_039838492.1	-	-	-	-	-	-	-

Table S3. Continued.

Class	Predicted function	Gene	Genome / Protein accession numbers											
			S. delphini NCTC 12225^T (dolphin)	*S. delphini* P5747 (penguin)	*S. delphini* P6456 (penguin)	*S. delphini* 215100905101-2 (horse)	*S. delphini* 8086 (horse)	*S. delphini* 14S03313-1 (horse)	*S. delphini* 14S03318-1 (marten)	*S. delphini* 14S03309-1 (pigeon)	*S. pseudintermedius* LMG 22219^T (cat)	*S. pseudintermedius* P8688 (seal)	*S. intermedius* NCTC 11048^T (pigeon)	*S. cornubiensis* NW1^T (human)
			GCF_900636325.1	GCF_009720305.1	GCF_009720295.1	GCF_002369695.1	GCF_000308115.1	GCF_002374125.1	GCF_002369645.1	GCF_002374115.1	GCF_001792775.2	GCA_009939245.1	GCF_900458545.1	GCF_900183575.1
Cell division	Cell division protein	*divIB*	WP_096596160.1	WP_155261695.1	WP_096596160.1	WP_096540591.1	WP_019165664.1	WP_096605182.1	WP_096545724.1	WP_096593096.1	WP_014614179.1	NCJ14699.1	SUM47016.1	WP_086429098.1
	Cell division initiation protein	*divIC*	WP_019165010.1 WP_096596156.1	WP_019165010.1	WP_019165010.1	WP_096543154.1	WP_019165010.1	WP_019165010.1	WP_019165010.1	WP_019165010.1	WP_014614798.1	NCJ13822.1	-	WP_086429490.1
	Autolysin	*lytA*	-	WP_155261051.1	WP_155259371.1	-	WP_019165358.1	-	-	WP_096661962.1	WP_015729543.1	NCJ14500.1	-	-
		lytD	WP_096596697.1	WP_019165956.1	WP_155259622.1	WP_096542040.1	WP_019165956.1	WP_096542040.1	WP_019165956.1	WP_019165956.1	WP_014613595.1	NCJ14010.1	SUM46414.1	WP_086427488.1
	N-acetylmuramoyl-L-alanine amidase	*lytH*	WP_096597105.1	WP_155261368.1	WP_155259718.1	WP_096541603.1	WP_019165151.1	WP_096541603.1	WP_096545132.1	WP_096592340.1	WP_014613729.1	NCJ15437.1	SUM46560.1	WP_086427615.1
Transporters	ABC transporter substrate-binding protein	-	WP_096598170.1	-	WP_155259279.1	WP_096542812.1	-	WP_096542812.1	WP_096544677.1	WP_096591046.1	WP_014613020.1	-	-	WP_086428022.1
		-	WP_096598178.1	-	WP_155259275.1	WP_096542822.1	-	WP_096606860.1	WP_096544687.1	-	WP_037542303.1	-	-	WP_086428017.1
		-	WP_096596403.1	WP_155260914.1	WP_155259199.1	WP_096541885.1	WP_019166312.1	-	WP_096544863.1	WP_096591186.1	WP_015729662.1	NCJ15159.1	SUM45751.1	WP_086429030.1
		-	-	WP_155260849.1	-	-	WP_019167021.1	-	WP_096546126.1	WP_096593542.1	-	-	SUM45660.1	WP_086428868.1
		troA	WP_096596571.1	-	WP_155260313.1	WP_096540292.1	WP_019166244.1	WP_096605036.1	WP_096544091.1	WP_096638128.1	WP_014614644.1	NCJ14123.1	-	WP_086428519.1
	Nickel ABC transporter	*opp-1A*	WP_096598140.1	WP_155260637.1	WP_155260437.1	WP_096539659.1	WP_019166928.1	WP_096604692.1	WP_096546233.1	WP_096591551.1	WP_014614880.1	NCJ15619.1	SUM45334.1	WP_086428762.1
	ABC transporter permease	-	WP_096597250.1	-	-	-	-	-	-	-	-	-	-	-
	Copper-translocating P-type ATPase	*zntA*	-	-	-	-	WP_019165821.1	-	-	-	-	-	-	-
	DMT family transporter	*ydcZ*	-	-	-	-	WP_019166257.1	-	-	-	-	-	-	-
	Phenylalanine-tRNA ligase subunit beta	-	-	-	WP_155260000.1	-	-	-	-	-	-	-	-	-
	Heavy metal translocating P-type ATPase	-	-	WP_155261165.1	-	-	-	-	-	-	-	-	-	-
	Divalent metal cation transporter	-	-	WP_155261384.1	-	-	-	-	-	-	-	-	-	-
Peptidases	Peptidase	-	WP_096597960.1	WP_155261166.1	WP_155259488.1	WP_096543009.1	WP_019165822.1	WP_096606408.1	WP_096547115.1	WP_096662133.1	WP_037542547.1	NCJ13872.1	SUM46158.1	WP_086429186.1
	Signal peptidase I	-	WP_096542133.1	WP_155262071.1	WP_155260188.1	WP_096542133.1	WP_019166008.1	WP_096542133.1	WP_096542133.1	WP_096661894.1	WP_014614397.1	NCJ13666.1	SUM47362.1	WP_086428973.1
	M23 family metallopeptidase	-	WP_096596442.1	WP_155260945.1	WP_155259230.1	WP_096542907.1	WP_026066938.1	-	WP_096544788.1	WP_026066938.1	-	NCJ15197.1	SUM45793.1	WP_086427975.1
		-	WP_096595998.1	-	-	WP_096539950.1	-	-	-	-	-	-	-	-
	Peptidase P60	-	-	-	-	-	-	-	-	-	WP_000768373.1	-	-	-
	Peptidase	-	WP_096597599.1	WP_155261198.1	-	-	-	WP_096606349.1	WP_096547250.1	-	WP_015729408.1	NCJ13837.1	-	-
	Peptidase T	*pepT*	-	-	WP_155260239.1	-	-	-	-	-	-	-	-	-
Other cell wall proteins	Peptidoglycan-binding protein	*lysM*	WP_096598096.1	WP_155262119.1	WP_155260419.1	WP_096539603.1	WP_019166377.1	WP_096604727.1	WP_096546301.1	WP_096557038.1	WP_014614841.1	NCJ15587.1	SUM45291.1	WP_086428731.1
	Competence protein	*comGC*	WP_096541451.1	WP_155261425.1	WP_096541451.1	WP_096541451.1	WP_019165232.1	WP_096541451.1	WP_096547032.1	WP_096541451.1	WP_015729165.1	NCJ15346.1	SUM46663.1	WP_086427699.1
	Dipeptide-binding protein	*oppA*	WP_096596536.1	WP_155261834.1	WP_155260158.1	WP_096542759.1	WP_019165989.1	WP_096604776.1	WP_096543577.1	WP_096592152.1	WP_020220047.1	NCJ13647.1	SUM47343.1	WP_086428955.1
	Kinase-associated lipoprotein B	*kapB*	WP_096596513.1	WP_155261853.1	WP_019166974.1	WP_019166974.1	WP_019166974.1	WP_096604822.1	WP_096543628.1	WP_019166974.1	WP_014614413.1	NCJ13686.1	SUM47376.1	WP_086428989.1
	Malate dehydrogenase (quinone)	*mqo2*	WP_096543297.1	WP_155260819.1	WP_096543297.1	WP_096595678.1	WP_019165095.1	WP_096543297.1	WP_096546063.1	WP_019165095.1	WP_014612817.1	NCJ15233.1	SUM45628.1	WP_086429552.1
	Malate-quinone-oxidoreductase 1	*mqo1*	WP_096597125.1	WP_155261011.1	WP_155259325.1	WP_096543189.1	WP_019165505.1	WP_096605528.1	WP_096544589.1	WP_096590975.1	WP_014613089.1	NCJ14563.1	SUM45897.1	WP_086428078.1
	Phosphodiesterase	-	WP_096596503.1	WP_019166993.1	WP_155260195.1	WP_096542188.1	WP_019166993.1	WP_096604838.1	WP_096543651.1	WP_096594020.1	WP_103263530.1	NCJ13709.1	SUM47400.1	WP_086429366.1
	Protein-disulfide isomerase	-	WP_096595914.1	WP_155260686.1	WP_155260490.1	WP_096539787.1	WP_019166051.1	WP_096604591.1	WP_096546722.1	WP_096591434.1	WP_096548384.1	NCJ15698.1	SUM45414.1	WP_086428837.1
	Ribonuclease H	-	WP_096596278.1	WP_155261483.1	WP_096554780.1	WP_096541138.1	WP_019165318.1	WP_096554780.1	WP_096545479.1	WP_096591828.1	WP_020219606.1	NCJ14945.1	SUM46753.1	WP_086427785.1
	Small heat shock protein	*hsp20*	WP_096596042.1	WP_155260800.1	WP_096596042.1	WP_019166846.1	WP_019166846.1	WP_096604418.1	-	WP_019166846.1	WP_015729757.1	NCJ15253.1	SUM45605.1	WP_086428680.1
	Thioredoxin-dependent thiol peroxidase	*bcp*	WP_096597706.1	WP_155261241.1	WP_155259578.1	WP_096543363.1	WP_019166484.1	WP_096606878.1	WP_096546362.1	WP_096592832.1	WP_014613508.1	NCJ15520.1	SUM46284.1	WP_086429233.1

Table S3. Continued.

Class	Predicted function	Gene	Genome / Protein accession numbers											
			S. delphini NCTC 12225^T (dolphin)	*S. delphini* P5747 (penguin)	*S. delphini* P6456 (penguin)	*S. delphini* 215100905101-2 (horse)	*S. delphini* 8086 (horse)	*S. delphini* 14S03313-1 (horse)	*S. delphini* 14S03318-1 (marten)	*S. delphini* 14S03309-1 (pigeon)	*S. pseudintermedius* LMG 22219^T (cat)	*S. pseudintermedius* P8688 (seal)	*S. intermedius* NCTC 11048^T (pigeon)	*S. cornubiensis* NW1^T (human)
			GCF_900636325.1	GCF_009720305.1	GCF_009720295.1	GCF_002369695.1	GCF_000308115.1	GCF_002374125.1	GCF_002369645.1	GCF_002374115.1	GCF_001792775.2	GCA_009939245.1	GCF_900458545.1	GCF_900183575.1
Other extracellular proteins	Chitosanase	*csn*	-	-	-	-	WP_019165731.1	-	-	-	-	-	-	WP_086428604.1
	CsbD-like protein	-	-	-	-	-	-	-	-	-	-	-	SUM43551.1	-
		-	WP_096541591.1	WP_155261373.1	WP_096541591.1	WP_096541591.1	-	WP_096541591.1	WP_096545141.1	WP_096545141.1	WP_014613736.1	NCJ15430.1	SUM46567.1	-
	DUF1307 domain-containing protein	-	-	WP_142294444.1	-	-	-	-	-	-	-	-	-	-
	Formate dehydrogenase subunit alpha	-	-	WP_155261050.1	-	-	-	-	-	-	-	-	-	-
	DUF669 domain-containing protein	-	-	-	-	-	-	-	-	-	-	-	SUM46349.1	-
	Subtilosin A	*sboA*	WP_096598771.1	-	-	-	-	-	-	-	-	-	-	-
	YolD-like family protein	-	-	-	-	-	-	-	-	-	WP_001798151.1	-	-	-
	Alpha/beta hydrolase	-	WP_096596067.1	WP_155260649.1	WP_155260447.1	WP_096540054.1	WP_083849004.1	WP_096604757.1	WP_096546349.1	WP_096662087.1	WP_063279062.1	NCJ15628.1	-	WP_086428865.1
	Amidase domain-containing protein	-	WP_096596398.1	WP_155260908.1	WP_155259195.1	WP_096541872.1	WP_019166305.1	WP_096605799.1	WP_096544871.1	WP_096661996.1	WP_103263650.1	NCJ15155.1	SUM45747.1	WP_086429025.1
	Carboxylesterase family protein	-	-	-	-	-	-	-	WP_142302633.1	-	-	-	-	-
	Deferrochelatase/ peroxidase	-	WP_096596559.1	WP_155261994.1	WP_019165102.1	WP_096595605.1	WP_019166265.1	WP_096595605.1	WP_096544122.1	WP_096661927.1	-	NCJ14102.1	SUM47619.1	WP_086428405.1
	DUF1722 domain-containing protein	-	-	WP_155261877.1	-	-	-	WP_096604866.1	-	-	-	-	-	-
	Adenosylmethionine-8-amino-7-oxononanoate transaminase	*bioA*	WP_096596024.1	-	-	-	-	-	-	-	-	-	-	-
	Cell fate regulator	*yaaT*	WP_096598399.1	-	WP_155260402.1	WP_096543167.1	-	-	-	-	-	-	-	-
	Hypothetical proteins	-	-	-	-	-	-	-	-	-	WP_103263459.1	NCJ14566.1	-	WP_086428075.1
		-	-	-	-	-	-	-	-	-	-	NCJ13602.1	-	-
		-	-	-	-	-	-	-	-	-	-	NCJ14457.1	-	-
		-	-	-	-	-	-	-	-	-	-	NCJ14406.1	-	-
		-	-	-	-	-	WP_026067011.1	-	-	-	-	-	-	-
		-	-	-	WP_155260499.1	-	-	-	-	-	-	-	-	-
		-	-	WP_155261267.1	-	-	-	-	-	-	-	-	-	-
		-	WP_096596966.1	-	WP_096541503.1	-	-	-	-	-	-	-	-	-
		-	WP_096598679.1	-	-	-	-	-	-	-	-	-	-	-
		-	WP_126489886.1	-	-	-	-	-	-	-	-	-	-	-
		-	-	WP_155262125.1	WP_155260084.1	WP_096595558.1	-	-	-	-	-	-	-	-
		-	-	-	-	-	-	-	WP_096543794.1	-	-	-	-	-
		-	-	-	-	-	-	-	WP_096545244.1	-	-	-	-	-
		-	-	-	-	-	-	WP_096606315.1	-	-	-	-	-	-
		-	-	-	-	-	-	-	-	WP_096662163.1	-	-	-	-
		-	-	-	-	-	-	-	-	WP_096662188.1	-	-	-	-
		-	-	-	-	-	-	-	-	-	-	-	-	WP_015728984.1
		-	-	-	-	-	-	WP_096606888.1	-	-	-	-	-	-
		-	-	-	-	-	-	-	-	-	WP_112424797.1	-	-	-
		-	-	-	-	-	-	-	-	-	-	-	SUM43753.1 SUM43646.1	-
		-	-	-	-	-	-	-	-	-	-	-	SUM43753.1 SUM43646.1	-
		-	-	-	-	-	-	-	-	-	-	-	SUM43779.1	-
		-	-	-	-	-	-	-	-	-	-	-	SUM43893.1	-
		-	-	-	-	-	-	-	-	-	-	-	SUM46387.1	-
		-	WP_096596082.1	WP_155261984.1	WP_155260049.1	WP_096542975.1	WP_019165887.1	-	-	-	-	-	SUM46568.1	-
		-	-	-	-	-	-	-	-	-	-	-	SUM46893.1	-
		-	-	-	-	-	-	-	-	-	-	-	SUM47179.1	-
